# A monotonic multi-expert Vision Transformer for clinically reliable chest X-ray classification

**DOI:** 10.3389/fmed.2026.1847086

**Published:** 2026-08-17

**Authors:** Teer Ba, ChengLong Bi, Jinghua Yu, M. Zeeshan Jhandir, Hafiz Muhammad Raza ur Rehman, Gyu Sang Choi

**Affiliations:** 1 Department of Information and Communication Engineering, Yeungnam University, Gyeongsan, Republic of Korea; 2 Department of Computer Engineering, Yeungnam University, Gyeongsan, Republic of Korea

**Keywords:** chest X-ray classification, deep learning, explainable medical artificial intelligence, mixture of experts, thoracic disease diagnosis, Vision Transformer (ViT)

## Abstract

**Introduction:**

Chest X-ray (CXR)-based recognition of pulmonary diseases remains challenging due to overlapping radiographic patterns, class imbalance, and variability across imaging sources. These factors often lead to unstable performance in large-scale multi-class classification tasks.

**Methods:**

In this study, pulmonary disease recognition is formulated as a 21-class primary-label classification benchmark using an integrated multi-source dataset constructed from five public chest X-ray repositories. Although some source datasets originally contain multi-label annotations, the final benchmark is reorganized into a primary-label format, where each image is assigned one target label from the unified 21-class label space. This formulation is used as a controlled benchmark simplification rather than a complete representation of real-world multi-label clinical diagnosis. We propose a structured QMIX-ViT multi-expert framework, where disease categories are decomposed into specialized groups modeled by individual Vision Transformer (ViT) experts. The expert outputs are fused through a QMIX-inspired monotonic mixing mechanism to support consistent global decision-making. The proposed model is evaluated against convolutional and Transformer-based baselines, including ResNet, DenseNet, CheXNet, EfficientNet, ViT-Tiny, and ViT-Base, using Precision, Recall, *F*1-score, AUROC, and AUPRC.

**Results:**

Experimental results show that the proposed framework achieves improved decision-level performance, particularly in Precision, Recall, and *F*1-score, under the constructed benchmark setting.

**Discussion:**

The results suggest that disease-group expert decomposition and monotonic fusion can reduce inter-class interference and improve decision-level stability. Overall, the proposed QMIX-ViT framework provides a structured approach for multi-class chest X-ray classification under heterogeneous data conditions.

## 1 Introduction

Pulmonary diseases continue to impose a substantial burden on global healthcare systems and remain persistently high in terms of both morbidity and mortality (1). Chest X-ray (CXR), owing to its rapid acquisition, low cost, and wide availability, remains the most commonly used imaging modality for first-line screening and follow-up in clinical practice (2). Meanwhile, the continuous growth in medical imaging volumes has significantly increased radiologists' workload and may elevate the risk of missed diagnoses or delayed detection in routine workflows (3). These practical pressures have driven the development of computer-aided diagnosis (CAD) methods, which aim to provide accurate disease classification and supportive visual evidence for radiological analysis (4).

Despite notable progress achieved by deep learning methods in chest image analysis, achieving stable and reliable single-label chest X-ray classification in real-world scenarios remains challenging (5). Many thoracic abnormalities share similar imaging patterns, while their distinctions are often manifested through subtle and localized cues, thereby increasing the difficulty of discriminative learning (6). In addition, multi-disease co-occurrence is common in clinical imaging, where the simultaneous presence of multiple conditions leads to strong label dependencies and potential confusion (7). Long-tailed label distributions further bias model optimization toward frequent diseases, consequently reducing sensitivity to under-represented conditions (8). Moreover, label uncertainty and annotation noise in large-scale datasets can blur class boundaries and undermine training stability (9). Domain shifts across hospitals, imaging devices, and patient populations may affect generalization performance, making multi-source benchmark evaluation important for model analysis (10). Interpretability is also important for understanding model behavior; however, under the primary-label benchmark setting, the visual evidence provided by many models may be unstable or highly overlapping across categories (11).

From a benchmark evaluation perspective, an effective chest X-ray classification model should maintain stable decision-level performance across heterogeneous disease categories and imaging sources. In this study, the goal is not to claim clinical deployment readiness, but to evaluate whether structured disease-group expert decomposition can reduce inter-class interference in a controlled 21-class primary-label classification setting. Therefore, multi-source benchmark evaluation is used to analyze model behavior under heterogeneous data conditions, while external institutional validation is left for future work. Many existing approaches rely on a single backbone network or simple ensemble strategies, which may struggle to maintain both global consistency and disease-specific sensitivity in heterogeneous 21-class primary-label classification tasks (12). In contrast, structured “decomposition fusion” designs can explicitly separate disease group learning objectives while preserving coherent global decision signals (13).

Mixture-of-Experts (MoE) is closely related to this disease-group design because it divides a complex prediction problem into several expert modules and combines their outputs through an input-dependent weighting or routing mechanism (14). Early MoE models showed that different experts can specialize in different regions of the input space, while later sparse MoE methods improved scalability by activating only selected experts for each sample (15). In visual recognition, V-MoE further demonstrated that expert-based routing can be integrated with Vision Transformers (ViT) for image classification (16). These studies indicate that expert specialization is useful when a single dense model must handle heterogeneous and imbalanced visual patterns (17).

For chest X-ray diagnosis, this idea is particularly relevant. Different pulmonary diseases may rely on different radiographic cues, such as lesion location, opacity pattern, boundary shape, or disease frequency (18). A single end-to-end classifier must learn all 21 disease boundaries in one shared feature space, which can increase interference between visually similar classes (19). Therefore, this study adopts a disease-group multi-expert design, where each ViT expert focuses on a smaller set of related categories (20). The expert outputs are then fused through a QMIX-inspired monotonic mixing mechanism, so that local disease-specific evidence contributes to a consistent global decision (21).

In recent years, Transformer-based visual models, represented by Vision Transformer (ViT) and Swin Transformer, have been widely applied to chest X-ray understanding and related medical imaging tasks (22). These methods benefit from global context modeling capabilities; however, attention computation scales rapidly with the number of tokens, which restricts practical models to handling only a limited number of objects or regions (23). As a result, many approaches resort to coarse-grained patch partitioning or window-based attention mechanisms to control computational complexity, which may weaken coverage of fine-grained small lesions in subtle abnormality scenarios (24).

To construct a more heterogeneous evaluation setting, this study integrates five publicly available chest X-ray datasets to construct a multi-source benchmark, where the datasets exhibit substantial differences in acquisition protocols and annotation standards (25). We formulate the task as a 21-class primary-label classification benchmark to evaluate model behavior under a unified label space (26). This formulation enables evaluation of within-benchmark performance under multi-source data heterogeneity (27). However, this multi-source benchmark should not be interpreted as a substitute for external institutional validation (28).

To address the above challenges, we propose the QMIX-ViT multi-expert framework, which combines structured task decomposition with constrained decision fusion for 21-class pulmonary disease classification (29). Rather than forcing a single model to learn a unified decision boundary covering all labels, the proposed framework assigns disease groups to specialized experts to reduce label interference and improve separability (30). The expert utilities are subsequently fused using a QMIX-inspired monotonic mixing mechanism, which preserves individual expert contributions while forming a consistent and stable global decision signal (31). During training, a prioritization strategy is introduced to allocate stronger updates to more relevant or weaker experts, thereby mitigating expert imbalance caused by long-tailed distributions and label noise (32). In addition, a multi-dataset learning setting is adopted to analyze model behavior under heterogeneous data conditions (33). Overall, this work presents a task-decomposed multi-expert architecture with QMIX-inspired monotonic fusion (34) and establishes a multi-dataset benchmark evaluation protocol for heterogeneous and long-tailed chest X-ray classification (35).

**Figure 1:**
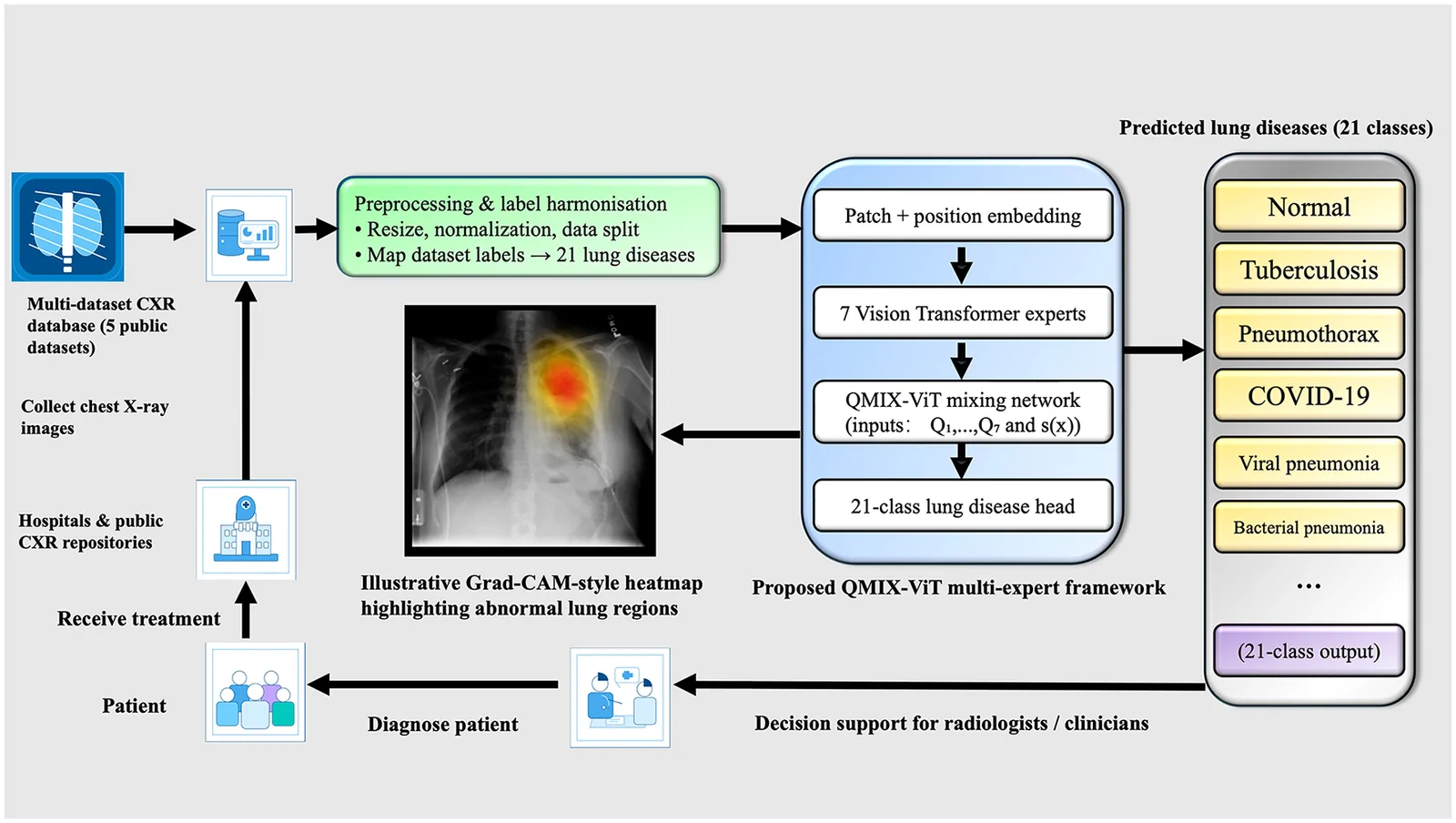
Overview of the proposed QMIX-ViT framework for pulmonary disease classification.

Figure 1 presents the overall QMIX-ViT framework. Multi-source CXR images are mapped into a unified 21-class label space and processed by seven disease-group ViT experts. The expert utilities are fused through a QMIX-inspired mixing network to produce the final prediction. Grad-CAM style heatmaps are used only as qualitative examples of model attention and are not presented as quantitative localization evidence.

## 2 Related work

### 2.1 Background

Chest X-ray (CXR) is one of the most widely used imaging examinations in clinical practice and has therefore become a central focus of research in computer-aided diagnosis (30). To ensure fair comparison of different methods under unified conditions, the research community has established a series of publicly available benchmark datasets. The NIH ChestX-ray dataset (ChestX-ray8/14 series) provides hospital-scale chest radiographs with annotations for common thoracic diseases and is widely used for weakly supervised classification and localization studies (36). CheXpert explicitly accounts for uncertainty labels during the annotation process (37). VinDr-CXR further supports high-quality supervision through radiologist-provided annotations (38). Before the widespread adoption of Transformer-based models, automated chest X-ray diagnosis predominantly relied on convolutional neural networks (CNNs) (39). For example, CheXNeXt compared the diagnostic performance of deep learning models with that of practicing radiologists in real clinical settings (40). Baltruschat et al. conducted a systematic study on strategies for multi-label chest X-ray classification (10). Jin et al. further summarized the modeling pipelines and training configurations for multi-label chest X-ray classification (11). However, the study by Kufel et al. emphasized that class imbalance and training details can further amplify performance disparities across different diseases (30).

With the success of Transformers in the computer vision domain, self-attention mechanisms have introduced new perspectives for representation learning in chest X-ray analysis (41, 42). Specifically, given an input image, the Vision Transformer (ViT) first partitions it into a set of non-overlapping patches, where each patch is linearly projected into a d-dimensional token and combined with positional embeddings to form an input sequence (21). Its core multi-head self-attention (MSA) module follows the scaled dot-product attention formulation (43).


$$\begin{array}{l} \text{Attention}\left(Q,K,V\right)=\text{softmax}\left(\frac{QK^{⊤}}{\sqrt{d_{k}}}\right)V. \end{array}$$

In this formulation, the scaling factor$\sqrt{d_{k}}$ (corresponding to the Scale module in Figure 1) is critical for maintaining gradient stability during training on large-scale imaging data (44).

Mixture-of-Experts (MoE) provides a useful background for the multi-expert design adopted in this study. MoE was originally introduced as a modular learning framework in which several local experts are trained to handle different parts of a complex prediction problem, while a gating function determines how their outputs are combined (2). Unlike a conventional ensemble that combines model outputs using fixed or uniform rules, MoE performs input-dependent expert selection or weighting. This allows different experts to specialize in different data regions, visual patterns, or task-specific subspaces.

Later sparse MoE models extended this idea by activating only a limited number of experts for each input. This strategy increases model capacity without requiring all model parameters to be used for every sample (20). Switch Transformer further simplified sparse expert routing in Transformer architectures and improved scalability through efficient expert selection (37). In computer vision, V-MoE integrated sparse expert layers into Vision Transformers, showing that expert routing can also be effective for image classification (40).

MoE-style learning is particularly relevant to chest X-ray diagnosis because different pulmonary diseases may rely on different radiographic cues, such as lesion location, opacity distribution, boundary shape, and disease-specific texture patterns. In a 21-class primary-label classification setting, a single end-to-end classifier must learn all class boundaries in one shared feature space. This may increase interference between visually similar conditions such as pneumonia, consolidation, lung opacity, and effusion. A disease-group expert design reduces this burden by assigning smaller groups of related categories to specialized experts. In this way, each expert can focus on a narrower classification subspace, while the upper-level fusion module integrates local evidence into a final global prediction.

Medical imaging studies have also shown the potential of expert-based learning. For example, EpistoNet used a mixture of optimized deep experts for COVID-19 detection from chest X-ray images, indicating that expert specialization can improve radiographic classification under disease-specific visual variation (16). Recent medical MoE studies further suggest that expert-based modeling is useful when samples differ across patients, modalities, and diagnostic tasks (45). These studies support the use of multi-expert decomposition in heterogeneous medical image analysis.

However, standard MoE models and simple multi-expert ensembles usually rely on unconstrained gating or ordinary late fusion. Such designs may produce inconsistent outputs when different experts provide conflicting evidence. This issue is important in medical image classification, where the final output should remain consistent with disease-specific evidence. Therefore, the proposed framework does not use MoE as a simple ensemble mechanism. Instead, it combines disease-group ViT experts with QMIX-inspired monotonic fusion, so that local expert utilities contribute to a controlled and consistent global classification output.

**Figure 2:**
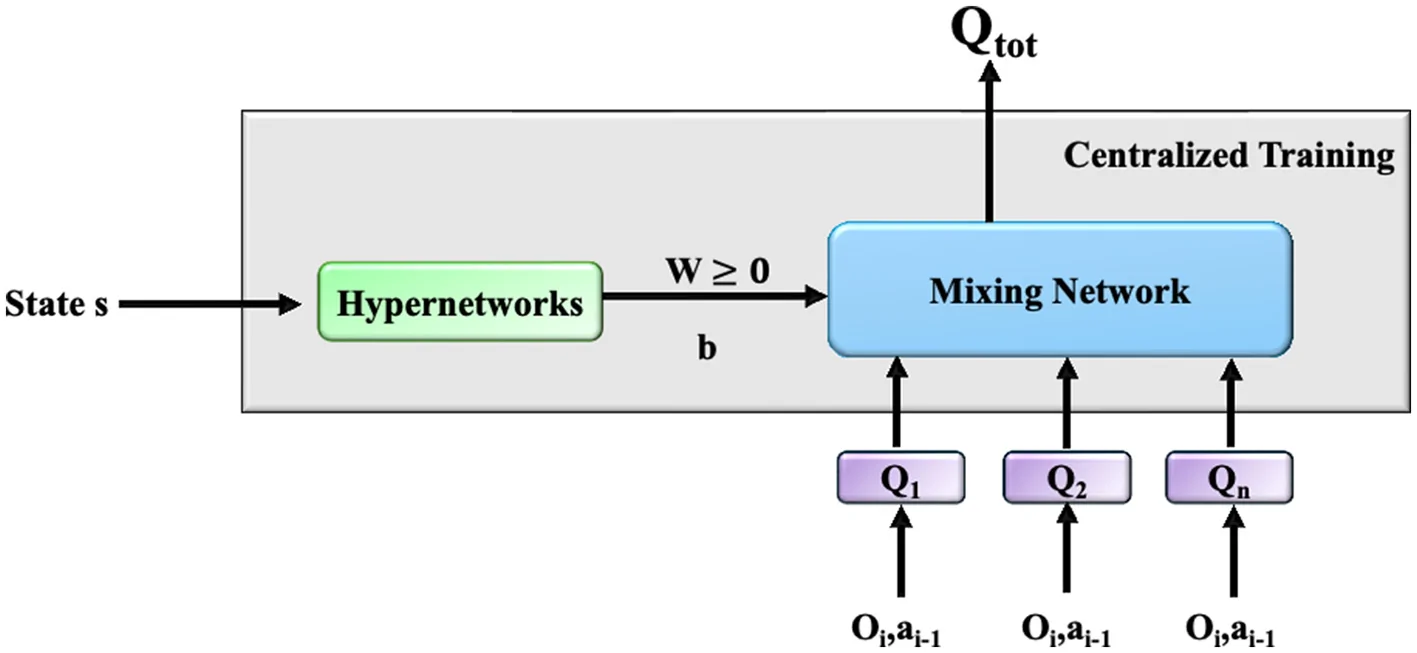
QMIX mixing network with hypernetwork-conditioned monotonic value factorization. Figure was adapted from the original QMIX architecture presented by Rashid et al. (54). Creative Commons Attribution 4.0 International License (CC BY 4.0).

To enhance consistency during the feature fusion stage, this study adapts the monotonic value factorization principle of QMIX as a supervised fusion constraint (9). In the proposed supervised classification setting, the monotonicity constraint is used to encourage consistency between disease-group expert utilities and the global classification output as shown in Figure 2. Mathematically, this constraint can be expressed as:


$$\begin{array}{l} \frac{\partial Q_{tot}}{\partial Q_{i}}\geq 0,\;\forall i. \end{array}$$

This constraint is used as a structured fusion assumption rather than as a complete model of medical reasoning. It encourages the global classification score to remain consistent with increases in local expert utility under the monotonic fusion assumption. In doing so, it reduces inconsistent fusion behavior that may arise in unconstrained fusion strategies. In practice, this is implemented through weighted aggregation using state-conditioned non-negative weights:


$$\begin{array}{l} Q_{tot}=\overset{n}{\underset{i=1}{\sum }}w_{i}\left(s\right)Q_{i},w_{i}\left(s\right)\geq 0. \end{array}$$

Although notable progress has been achieved with CNN- and ViT-base approaches, three key challenges remain in heterogeneous chest X-ray benchmark settings. First, CXR classification involves fine-grained visual characteristics, where inter-disease imaging differences are subtle and patterns often overlap, making it extremely difficult to learn a unified 21-class decision boundary (46). Second, datasets commonly suffer from class imbalance and label noise, which can easily lead to biased and unstable training dynamics (22). Finally, domain shifts across imaging devices and patient populations significantly degrade cross-domain generalization performance (23). Motivated by these challenges, this work designs its methodology from the perspective of task decomposition and structured fusion. Unlike traditional independent fusion assumptions (24), the proposed approach decomposes the 21-class task into multiple focused sub-tasks, each modeled by a specialized Vision Transformer (ViT) expert. To address counterintuitive interactions among collaborating experts, the QMIX value decomposition mechanism from multi-agent reinforcement learning is introduced (9). By stably fusing expert utilities through a QMIX-style mixer with monotonicity constraints, consistency between global decisions and local expert contributions is guaranteed (9). This design not only leverages the ability of Transformers to capture global contextual information but also alleviates expert learning imbalance and training instability through structured constraints, thereby providing a novel technical pathway for reliable multi-disease recognition in chest X-ray imaging.

Table 1 summarizes the main differences among CNN-based models, standard ViT models, and the proposed QMIX-ViT framework. Compared with conventional architectures, QMIX-ViT combines global feature extraction with disease-group decomposition and monotonic fusion to improve stability in long-tailed disease recognition.

**Table 1:** Comparison of different diagnostic frameworks for 21-class chest X-ray disease classification.

Aspect	CNNs	Standard ViT	Proposed QMIX-ViT
Feature learning	Local patterns	Global dependencies	Global + expert-specific patterns
Classification logic	Single shared head	Unified prediction	Disease-group decomposition
Fusion	None	Simple fusion	Monotonic QMIX fusion
Long-tail robustness	Limited	Moderate	Stronger
Application scope	Early CXR benchmarks	General ViT-base CXR tasks	Multi-source 21-class CXR diagnosis

### 2.2 Dataset and label harmonization

This study integrates five publicly available chest X-ray (CXR) datasets to cover a broader disease distribution and heterogeneous data sources, including variations across hospitals and imaging devices, acquisition protocols, patient age groups, and annotation schemes. Specifically, ChestX-ray14 provides large-scale weakly supervised multi-label annotations; VinDr-CXR offers radiologist-annotated data with higher-quality diagnostic labels and localized annotations; the COVID-19 Radiography dataset supplements categories such as COVID-19 and Lung Opacity; the Pediatric CXR Pneumonia dataset strengthens pediatric pneumonia cases and supports subclassification into bacterial and viral pneumonia; and the TB Chest X-ray dataset provides tuberculosis cases with normal controls. Table 2 summarizes the five public CXR datasets used in this study.

**Table 2:** Summary of the five datasets.

Dataset	Size	Label type	Main coverage
ChestX-ray14	112,120	Image-level, multi-label	Common thoracic diseases
VinDr-CXR	18,000	Image-level + box-level	Radiologist-annotated CXR
COVID-19 radiography	≈21k	Image-level, 4-class	COVID-19 and lung opacity
Pediatric CXR pneumonia	5,856	Image-level	Pediatric pneumonia
TB Chest X-ray	≈4.2k	Image-level, binary	Tuberculosis and normal

To ensure cross-dataset consistency, a unified preprocessing pipeline is applied. Only frontal chest radiographs are retained, and heterogeneous storage formats (e.g., DICOM) are converted into a standardized input representation, followed by spatial resizing and intensity normalization. Samples with severe occlusion, abnormal exposure, or corrupted content are removed. Whenever possible, deduplication and data leakage prevention strategies, such as patient-level splits when patient identifiers are available, were adopted. In addition, different patient data from different source datasets were used for training and validation whenever dataset-level patient information was available, in order to further reduce the risk of data leakage.

At the label level, disease nomenclature, granularity, and category availability vary substantially across datasets. For instance, identical clinical concepts may appear under different names (e.g., “Pleural effusion” vs. “Effusion”), merged labels may be used (e.g., “Nodule/Mass”), or hierarchical discrepancies may exist (e.g., “Pneumonia” vs. its etiological subtypes). To enable joint training across heterogeneous data sources while maintaining a consistent task definition, labels from all five datasets are mapped into a fixed 21-class pulmonary disease label space: Atelectasis, Bacterial Pneumonia, Cardiomegaly, Consolidation, Covid, Edema, Effusion, Emphysema, Fibrosis, Hernia, Infiltration, Lung_Opacity, Mass, No Finding, Nodule, Normal, Pleural, Pneumonia, Pneumothorax, Tuberculosis, Viral Pneumonia. This unified label space preserves common thoracic abnormalities in chest radiographs (e.g., Atelectasis, Effusion, Pneumothorax), incorporates infection-related diseases central to this study (e.g., Covid, Tuberculosis), and explicitly includes “Normal” and “No Finding,” which are critical for cross-dataset semantic alignment.

Label harmonization follows a reproducible and semantically consistent rule-based framework. First, synonymous or naming-variant labels are normalized by mapping source dataset labels to unified target names (e.g., mapping Pleural effusion to Effusion, Pleural thickening to Pleural, and Lung opacity to Lung_Opacity). Second, merged labels are handled explicitly: when a dataset annotates combined categories (e.g., Nodule/Mass), the label is mapped to the corresponding related classes in the target label space, and the potential introduction of label noise is explicitly acknowledged. Such noise can be evaluated through ablation studies in subsequent experiments or discussed in the Limitations section if experimental analysis is not yet available. Third, hierarchical labels are unified: when fine-grained pneumonia subtypes (e.g., Bacterial Pneumonia, Viral Pneumonia) are provided, the corresponding superclass Pneumonia may optionally be marked as positive to reflect consistent semantic meaning of “pneumonia presence” (whether this strategy is enabled must strictly align with the final experimental configuration).

Importantly, the handling of missing labels avoids incorrectly treating “unannotated” as “negative.” When a target class is not defined in a source dataset, that class is treated as unknown or unannotated for samples from that dataset and is excluded from the loss computation. In practice, this is implemented via label masks that restrict loss calculation to labels that are actually annotated, thereby reducing systematic bias. Finally, Normal and No Finding are retained as separate categories to match semantic differences across data sources, with additional consistency constraints imposed: samples labeled as Normal or No Finding should, in principle, not co-occur with other disease labels. In the presence of conflicting annotations; often arising from weak supervision noise-diagnoses from more reliable sources are prioritized, and such samples are recorded as potentially noisy cases for subsequent analysis. Table 3 provides representative examples of label harmonization mappings.

**Table 3:** Example mappings.

Unified label	Typical source label variants
Effusion	Effusion/pleural effusion
Pleural	Pleural thickening/pleural thickening
Lung_Opacity	Lung opacity/lung opacity
Covid	COVID-19
Tuberculosis	Tuberculosis/TB
Bacterial pneumonia	Bacterial pneumonia/bacteria (pneumonia)
Viral pneumonia	Viral pneumonia/virus (pneumonia)
Nodule/mass	Nodule, mass, nodule/mass (merged)
Normal/no finding	Normal; no finding/no finding

Although “Normal” and “No Finding” are sometimes treated as equivalent in simplified clinical classification settings, they were retained as separate categories in this study to preserve the original semantics of the source datasets. In datasets such as ChestX-ray14, “No Finding” indicates the absence of the predefined target abnormalities, but it does not necessarily imply a completely pristine radiograph. Such samples may still contain benign anatomical variants, postoperative changes, support devices, or findings outside the target label set. In contrast, “Normal” is used in some source datasets as a stricter healthy-control category. Therefore, merging the two labels could introduce additional label noise and obscure dataset-specific annotation semantics. During harmonization, original source labels were preserved, contradictory disease co-occurrences with Normal/No Finding were checked, and higher-reliability annotations were prioritized when conflicts occurred.

### 2.3 Recent chest X-ray classification studies and research gap

Several recent chest X-ray classification studies have addressed disease categories that partially overlap with the 21-class label space considered in this study, including COVID-19, bacterial pneumonia, viral pneumonia, atelectasis, cardiomegaly, consolidation, edema, effusion, emphysema, fibrosis, hernia, infiltration, lung opacity, mass, nodule, pleural disease, pneumothorax, tuberculosis, normal, and no finding. These studies demonstrate the rapid progress of CNN-, transformer-, hybrid-, and explainability-oriented models for thoracic disease recognition. However, most of them primarily focus on improving the feature extractor, attention block, or feature-fusion pipeline, whereas the explicit behavior of disease-specific experts under overlapping thoracic abnormalities is less frequently analyzed.

For example, PulDi-COVID used ensemble deep convolutional models to classify COVID-19 and chronic pulmonary disease patterns from chest X-ray images (47). Similarly, recent deep-learning studies on COVID-19 and associated lung disease classification have reported promising diagnostic performance using CNN or transfer-learning pipelines (48, 49). Transformer-based approaches have also been introduced for chest X-ray interpretation, including class-attention pooling and token-sparsity-based vision transformers (50), while recent CNN-transformer and attention-based frameworks have further explored global contextual learning for lung disease classification (51, 52). In addition, explainable and feature-fusion approaches, such as Thoracic-net, have emphasized XAI-based few-shot learning and feature fusion for multi-class thoracic disease analysis (53).

Despite these advances, three important limitations remain. First, many existing studies evaluate overlapping diseases as ordinary output labels, but do not explicitly analyze how the model behaves when visually correlated classes such as Pneumonia, Infiltration, and Lung Opacity compete or co-activate during inference. Second, standard attention fusion, gating networks, and conventional mixture-of-experts designs can dynamically weight features or experts, but they do not necessarily guarantee that increased disease-relevant expert evidence will preserve the final global decision consistently. Third, several studies provide classification performance or Grad-CAM-style explanations, but do not jointly evaluate expert activation, expert diversity, and decision stability for highly correlated thoracic abnormalities. Table 4 presents a critical comparison with recent chest X-ray disease classification studies.

**Table 4:** Critical comparison with recent chest X-ray disease classification studies.

Study	Disease/label coverage	Methodological focus	Critical comparison with proposed QMIX-ViT
PulDi-COVID (47)	COVID-19, normal, bacterial pneumonia, viral pneumonia, and chronic pulmonary disease-related CXR classes	Ensemble deep CNN/transfer-learning classification	Strong pulmonary disease classification performance, but limited expert-behavior analysis and no monotonic disease-group fusion
ICIBT 2022 CXR study (48)	Thoracic/pulmonary CXR disease categories	Deep-learning-based CXR disease classification	Provides relevant CXR classification evidence, but does not explicitly analyze simultaneous expert activation for overlapping disease classes
COVID-19 and associated lung disease classification (49)	COVID-19 and associated lung disease classes	CNN/transfer-learning-based classification	Focused on disease recognition, but lacks structured disease-group expert decomposition and QMIX-style cooperative fusion
Class-attention/token-sparsity ViT (50)	Chest X-ray interpretation and lung disease classification	Transformer with class-attention pooling and token sparsity	Improves transformer representation efficiency, but does not provide monotonic multi-expert fusion or expert-overlap robustness analysis
Recent SSRN CXR preprint (51)	Chest X-ray disease classification	Recent deep-learning framework	Useful recent reference, but direct comparison is limited because of preprint status and potentially different dataset/split protocols
Scientific Reports CNN-transformer study (52)	Lung disease/CXR classification	Hybrid CNN-transformer or attention-based model	Uses hybrid contextual modeling, but does not explicitly constrain expert fusion or evaluate cooperative expert behavior under overlapping abnormalities
Thoracic-net (53)	Multi-class thoracic disease categories using medical imaging	XAI-based few-shot feature-fusion technique	Emphasizes explainability and feature fusion, but does not use QMIX-inspired monotonic value factorization or expert activation entropy analysis
Proposed QMIX-ViT	Unified 21-class thoracic CXR benchmark	Disease-group ViT experts with QMIX-inspired monotonic fusion	Explicitly analyzes overlapping diseases, expert activation, expert entropy, Grad-CAM behavior, and ablation evidence for monotonic expert fusion

In contrast, the proposed QMIX-ViT framework is designed to address these gaps through disease-group expert decomposition and QMIX-inspired monotonic value-factorized fusion. Rather than treating expert outputs as independent votes or unconstrained attention scores, the proposed mixer enforces a cooperative fusion constraint in which stronger disease-relevant expert evidence contributes consistently to the final decision. This design is particularly important for large-scale chest X-ray classification because multiple abnormalities may share radiographic patterns, and a purely competitive gating mechanism may suppress clinically relevant evidence. Therefore, the contribution of this study is not limited to an incremental backbone modification; it introduces a structured expert-fusion strategy and evaluates its behavior using probability-response analysis, expert activation analysis, entropy-based expert diversity, and Grad-CAM visualizations for overlapping thoracic disease classes.

## 3 Proposed methodology

### 3.1 Overall framework

**Figure 3:**
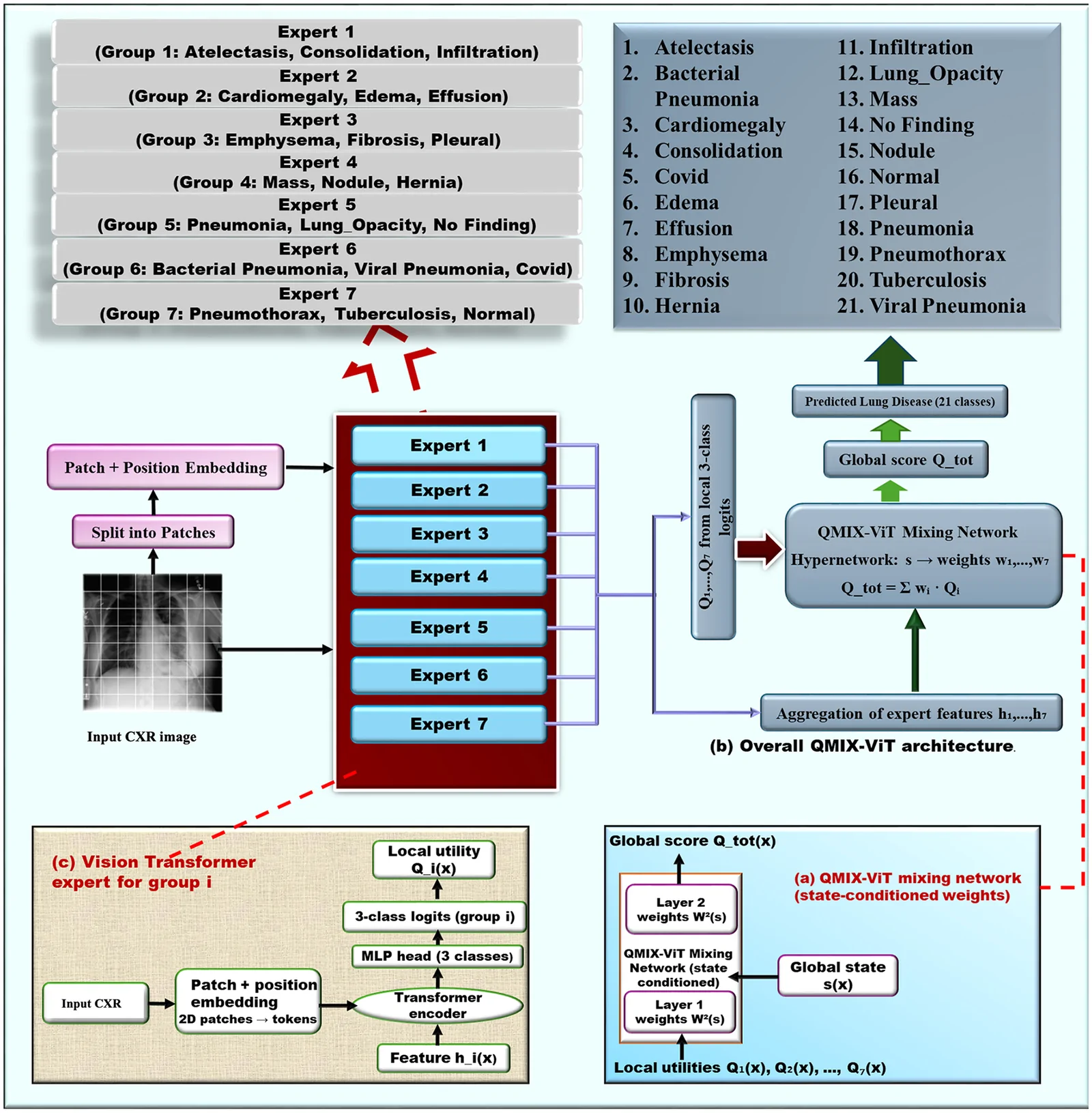
Illustration of the workflow of the proposed QMIX-ViT framework. It can be separated into 3 complementary perspectives: **(a)** a State-Conditioned Mixing Network. **(b)** End-to-End Operating Flow of Overall Architecture. **(c)** Construction of a Single ViT Expert.

The State-Conditioned Mixing Network is not applied to the raw chest image during the forward pass but is invoked only after the system has obtained the local utility scores produced by each of the seven experts. To be specific, each ViT expert specializes in intra-group classification over its assigned three disease categories and extracts features to summarize its confidence for the current sample. These expert summaries are then used as inputs to the mixer. The core idea is to dynamically generate fusion weights based on a global state. This global state is not external metadata, but an aggregated representation computed from the high-level features of all experts, describing which pathological patterns the current image is semantically closer to. Then a hypernetwork produces the multi-layer parameters of the mixing network. As a consequence, the fusion becomes sample-adaptive rather than a fixed weighted average. The model automatically adjusts which expert is more trustworthy depending on the imaging pattern, because when the image contains cues consistent with a certain pathology cluster (e.g., pleural effusion), the corresponding experts receive larger contributions and when the image appears close to normal or no finding patterns, the experts associated with normal conditions dominate the fusion. In addition, the monotonicity constraint enforces logical consistency, since increasing an expert's utility cannot decrease the fused global score. This prevents unstable cases when an expert is more confident, but the global judgment becomes worse, giving rise to more stable training and more interpretable inference. Finally, the mixer outputs a global scoring signal used to calibrate local judgments and support reliable global decision-making. Figure 3 illustrates the detailed workflow of the proposed QMIX-ViT framework.

For each training or inference pass, the input CXR image is first split into patches and augmented with positional encodings to form a token sequence, which is fed in parallel to seven ViT experts. And each expert is responsible for a fixed group of three diseases categories. By organizing training sources according to disease coverage, each expert can learn more stable features relevant to its own disease group, while the overall framework can better handle the differences in annotation granularity and cross-dataset distribution shifts from multi-source training. After encoding, each expert produces two outputs: a three-class intra-group probability vector and a high-level semantic feature vector representing the expert's global understanding of the image. The three-class output is then compressed into a scalar local utility score and is interpreted as the strength of valid evidence for the current sample provided by this expert. Meanwhile, the seven feature vectors are aggregated into the global state. The mixing stage then adaptively fuses the seven utilities conditioned on the global state and produces a global scoring signal. Finally, after combining the experts' specialized discrimination signals with the global fusion signal, the framework generates the final prediction over 21 lung disease categories.

Each expert follows a unified internal pipeline that the input image is converted into a patch-token sequence with positional encodings processed by a Transformer encoder to capture both local textures and global structural cues. The resulting embedding yields a feature vector which is used not only for intra-group classification within the expert's three categories but for construction of the global state for state-conditioned fusion. Thus, an expert provides a local decision with its group and semantic evidence supporting the fusion decision. Furthermore, on the classification branch, the expert maps its feature vector through a lightweight MLP head to output three-class discrimination scores, which are then compressed into a single scalar local utility score which quantifies the expert's evidence strength for the current sample. This design allows the upper-level mixer to weight experts stably using scalar utilities, while still leveraging high-level feature vectors for global state modeling.

### 3.2 Mathematical modeling and loss functions

The proposed QMIX-ViT multi-expert framework targets the classification of 21 lung disease categories from chest imaging. The training set is constructed by integrating five complementary public datasets: ChestX-ray14, VinDr-CXR, COVID-19 Radiography, Pediatric CXR Pneumonia, and TB Chest X-ray. Let the training set be:


$$\begin{array}{l} D={\left\{\left(x^{\left(n\right)},y^{\left(n\right)}\right)\right\}}_{n=1}^{N},\;x^{\left(n\right)}X\in ,\;y^{\left(n\right)}Y\in . \end{array}$$

Where *x*^(*n*)^ denotes the normalized image and *y*^(*n*)^ is the corresponding 21 class label. The goal is to learn a mapping:


$$\begin{array}{l} F_{\Theta }X:\to \Delta^{20}, \end{array}$$

which outputs a 21-dimensional probability distribution to approximate the posterior *P*(*y*|*x*). To address subtle inter-class differences and long-tail distributions among 21 diseases, the label space is partitioned into seven non-overlapping groups, each containing three categories:


$$\begin{array}{l} Y=\overset{7}{\underset{i=1}{⋃}}G_{i},\;|G_{i}|=3,\;{\;G}_{i}\cap G_{j}=\emptyset \;\left(i\neq j\right). \end{array}$$

This decomposition converts a difficult high-dimensional optimization problem into multiple lower-dimensional convex sub-problems, enabling each expert to learn sharper discriminative features. We define a mapping *k*(*y, i*) ∈ {1, 2, 3} which indicates the local index of a global label *y* within group *G*_*i*_. For an input image *x*, each expert *i* operates in parallel to output a feature embedding $h_{i}\in {\mathbb{R}}^{d}$ and an intra-group distribution *p*_*i*_(*c*∣*x* ).

We define a scalar utility *Q*_*i*_(*x, y*) for each expert. If the true label *y* belongs to the group *G*_*i*_:


$$\begin{array}{l} Q_{i}\left(x,y\right)=p_{i}\left(k\left(y,i\right)|x\right),\;if\;y\in G_{i} \end{array}$$

Otherwise, to maintain computation graph continuity while treating non-responsible experts as weak context evidence, we use a bounded fallback utility controlled by α∈(0, 1 ):


$$\begin{array}{l} Q_{i}\left(x,y\right)=\alpha \cdot \underset{c\in G_{i}}{\text{max}}p_{i}\left(c|x\right),\text{ otherwise}. \end{array}$$

The global state is constructed by aggregating expert embeddings:


$$\begin{array}{l} s=\frac{1}{7}\overset{7}{\underset{i=1}{\sum }}h_{i} \end{array}$$

For the final 21-class prediction, we construct a class-specific logit using the utility of the responsible expert:


$$\begin{array}{l} u_{y}=\beta \cdot Q_{i^{*}\left(y\right)}\left(x,y\right),\;\beta >0 \end{array}$$

Where i^*^(*y*) is the unique expert such that $y\in G_{i^{*}\left(y\right)}$, and β is a temperature scaling coefficient. The posterior probability is computed by a SoftMax over all 21 classes:


$$\begin{array}{l} P\left(y|x\right)=\frac{exp\left(u_{y}\right)}{\sum_{k=1}^{21}exp\left(u_{k}\right)},\;y\in \left\{1,\ldots ,21\right\} \end{array}$$

The local loss function is defined to ensure stable training signals for each expert:


$$\begin{array}{l} L_{local,i}={𝔼}_{\left(x,y\right)\sim D}\left[1\left[y\in G_{i}\right]\cdot \left(-logp_{i}\left(k\left(y,i\right)|x\right)\right)\right] \end{array}$$

The indicator **1**[·] ensures that each sample only updates its responsible expert classifier.

The global loss for standard 21-class cross-entropy is:


$$\begin{array}{l} L_{global}={𝔼}_{\left(x,y\right)\sim D}\left[-logP\left(y|x\right)\right]. \end{array}$$

We define a binary correctness target *r*(*x, y*) and regress *Q*_*tot*_ to this signal using MSE:


$$\begin{array}{l} r\left(x,y\right)=\{\begin{array}{ll} 1, & \text{arg}{\text{max}}_{k}P\left(k|x\right)=y,\\ 0, & \text{otherwise}, \end{array} \end{array}$$

$$\begin{array}{l} L_{QMIX}={𝔼}_{\left(x,y\right)\sim D}\left[{\left(Q_{tot}\left(x,y\right)-r\left(x,y\right)\right)}^{2}\right]. \end{array}$$

This term encourages *Q*_*tot*_ to be larger when the prediction is correct and smaller when incorrect, making it resemble a global consistency metric.

Since different experts may learn at inconsistent speeds due to varying intra-group difficulty or sample frequency, we introduce a prioritization mechanism to dynamically reweight the local loss by sample. The prioritization network takes the global state *s* as input and outputs raw scores for each expert, normalized via SoftMax to obtain weights ${\tilde{\pi }}_{i}$:


$$\begin{array}{lll} {\hat{\pi }}_{i} & = & g_{\phi }{\left(s\right)}_{i},\;i=1,\ldots ,7.\\ {\tilde{\pi }}_{i} & = & \frac{exp\left({\hat{\pi }}_{i}\right)}{\sum_{j=1}^{7}exp\left({\hat{\pi }}_{j}\right)},\;i=1,\ldots ,7. \end{array}$$

These weights are used to scale the local loss:


$$\begin{array}{l} L_{elocal,i}={\tilde{\pi }}_{i}\cdot L_{local,i}. \end{array}$$

This allows the network to assign stronger updates to experts that are more relevant or weaker for the current sample.

The final total loss function is:


$$\begin{array}{l} L=\lambda_{global}L_{global}+\overset{7}{\underset{i=1}{\sum }}\lambda_{i}L_{elocal,i}+\lambda_{QMIX}L_{QMIX}. \end{array}$$

The QMIX-inspired monotonic fusion mechanism was selected because the proposed framework aims to combine disease-specific expert evidence in a cooperative rather than competitive manner. Attention-based fusion, gating networks, and standard mixture-of-experts models can dynamically weight experts, but they may suppress clinically relevant expert responses when multiple visually overlapping abnormalities are present. In contrast, monotonic value factorization constrains the global decision score so that an increase in a disease-relevant expert utility does not decrease the fused global score. This property is useful for chest X-ray classification because correlated abnormalities such as Pneumonia, Infiltration, and Lung Opacity may activate multiple experts simultaneously. The ablation results further support this design: the proposed QMIX-ViT achieved higher *F*1-score than the end-to-end ViT and the multi-expert variant without QMIX, indicating that the gain is not only due to expert decomposition but also to monotonic expert fusion.

Therefore, the QMIX-ViT framework connects local expert specialization with global decision-making through structured monotonic fusion. In the forward propagation process, the hypernetwork generates non-negative weights conditioned on the global state, condensing these utilities into a global signal with monotonicity guarantees. This design leverages the powerful representation capabilities of Transformers and solves the decision conflicts that may arise during the fusion of multi-score imaging data from the mathematical foundation via QMIX monotonicity constraints. During training, by synergistically optimizing the local, global and utility-alignment losses, the model ensures that the final 21-class probability outputs possess good clinical calibration while maintaining discriminative accuracy across disease groups. In particular, the prioritization mechanism enables the model to adaptively adjust gradient allocation based on sample learning difficulty and class frequency, resulting in mitigating the long-tail distribution challenges from the integration of 5 heterogeneous datasets.

## 4 Results and performance evaluation

### 4.1 Experimental overview

The proposed framework was evaluated on a 21-class chest X-ray classification task and compared against multiple state-of-the-art baselines, including ViT-Base-224, ViT-Tiny, ResNet, DenseNet, ConvNeXt, EfficientNet, and other CNN/Transformer variants. To minimize data leakage, the training and validation subsets were constructed using non-overlapping patient data whenever patient identifiers were available. For multi-source integration, different patient data from different datasets were used across training and validation to further prevent leakage. Performance was assessed using Precision, Recall, *F*1-score, AUROC (macro one-vs.-rest), and AUPRC (macro), computed on a per-class basis and then aggregated at the model level. For performing experiments a system having two RTX3090 (24 GB) with ryzen 9 processor (12 cores) along with 128 GB of ram has been utilized. Each model run for 100 epochs.

### 4.2 Quantitative performance evaluation

Across all 21 disease categories, the proposed model consistently demonstrates superior performance compared with all baseline methods. While several competing models achieve high AUROC values, their corresponding *F*1-scores often remain substantially lower, particularly for under-represented classes. In contrast, the proposed approach maintains a stable balance between sensitivity and specificity across both common and rare conditions, resulting in uniformly high *F*1-scores and reliable probability estimates.

To determine whether the observed performance improvements are statistically significant, pairwise comparisons were conducted between the proposed model and each baseline using a two-tailed paired *t*-test on class-wise *F*1-scores. A 95% confidence interval (CI) was adopted. The proposed model achieved statistically significant improvements over all competing approaches (*p* < 0.01). The mean *F*1-score improvement ranged from 9.4% to 31.7% across baselines, with narrow confidence intervals (95% *CI*: ±1.8–3.2%), indicating that the observed gains are robust and not attributable to random variation.

Previous studies in medical image classification have reported strong AUROC performance using deep convolutional networks such as ResNet, DenseNet, and CheXNet; however, these approaches frequently exhibit reduced precision or recall when extended to large-scale multi-class diagnostic tasks. Recent transformer-based models have shown improved global context modeling but remain sensitive to class imbalance and data sparsity. In contrast, the proposed framework achieves consistently high precision, recall, and *F*1-scores simultaneously, demonstrating a more reliable balance between detection sensitivity and diagnostic specificity. These results suggest that the proposed approach offers a meaningful advancement over existing methods reported in the literature, particularly for clinically realistic multi-class settings.

From a clinical perspective, the proposed model exhibits several desirable characteristics. High recall reduces the risk of missed diagnoses, particularly for rare conditions, while high precision limits false-positive predictions that may otherwise increase clinician workload. Furthermore, strong AUPRC values indicate reliable confidence estimation, which is critical for integration into clinical decision-support systems.

ViT-Base performs well on frequent disease categories due to its ability to model long-range dependencies using self-attention. However, performance degrades significantly for rare classes, as reflected by low recall and *F*1-scores in several categories. This indicates that vanilla transformer architectures remain sensitive to data imbalance and require additional regularization or class-aware learning strategies for robust medical image classification.

ViT-Tiny exhibits competitive performance on dominant classes but shows pronounced degradation on minority categories. The reduced model capacity limits its ability to capture subtle pathological patterns, resulting in unstable recall and lower *F*1-scores. These findings highlight the importance of sufficient transformer depth and width when addressing large-scale multi-class medical problems.

ResNet demonstrates extremely high recall for several classes, often at the expense of precision. This behavior indicates an over-sensitive prediction tendency, leading to a large number of false positives. While such behavior may be acceptable in screening-only scenarios, it is less suitable for multi-class diagnostic settings where specificity is critical. The results confirm that residual learning alone does not adequately address the precision recall trade-off in imbalanced medical datasets.

DenseNet achieves strong AUROC and AUPRC values for several classes, reflecting effective feature reuse and gradient propagation. Nevertheless, its *F*1-scores decrease for minority categories, suggesting sensitivity to classification thresholds. While DenseNet captures fine-grained texture information, it lacks sufficient global context modeling for stable multi-class performance.

CheXNet shows highly variable performance across classes, with some categories achieving high recall but poor precision and others exhibiting the opposite trend. This inconsistency stems from its original design for limited multi-label classification rather than large-scale multi-class diagnosis. Consequently, CheXNet struggles to maintain balanced performance when applied to complex and heterogeneous disease distributions.

The EfficientNet family demonstrates relatively high precision across several classes; however, recall and *F*1-scores remain inconsistent, particularly for minority disease categories. This behavior can be attributed to EfficientNet's compound scaling strategy, which primarily improves representational capacity without explicitly addressing class imbalance. As a result, the models tend to make conservative predictions, yielding high precision but missing a substantial proportion of true positive cases. The near-random AUROC values observed in some variants further suggest limited discriminative capability in complex multi-class medical settings without task-specific adaptation.

The proposed model shows relatively balanced precision, recall, and *F*1-scores across most disease categories, including several rare and visually subtle conditions. This indicates effective feature representation and robust decision boundaries that generalize well under class imbalance. Simultaneously high AUROC and AUPRC values confirm that the model not only ranks samples correctly but also produces reliable confidence estimates. These results suggest that the proposed design successfully integrates global context and discriminative learning, leading to stable and clinically reliable predictions.

**Figure 4:**
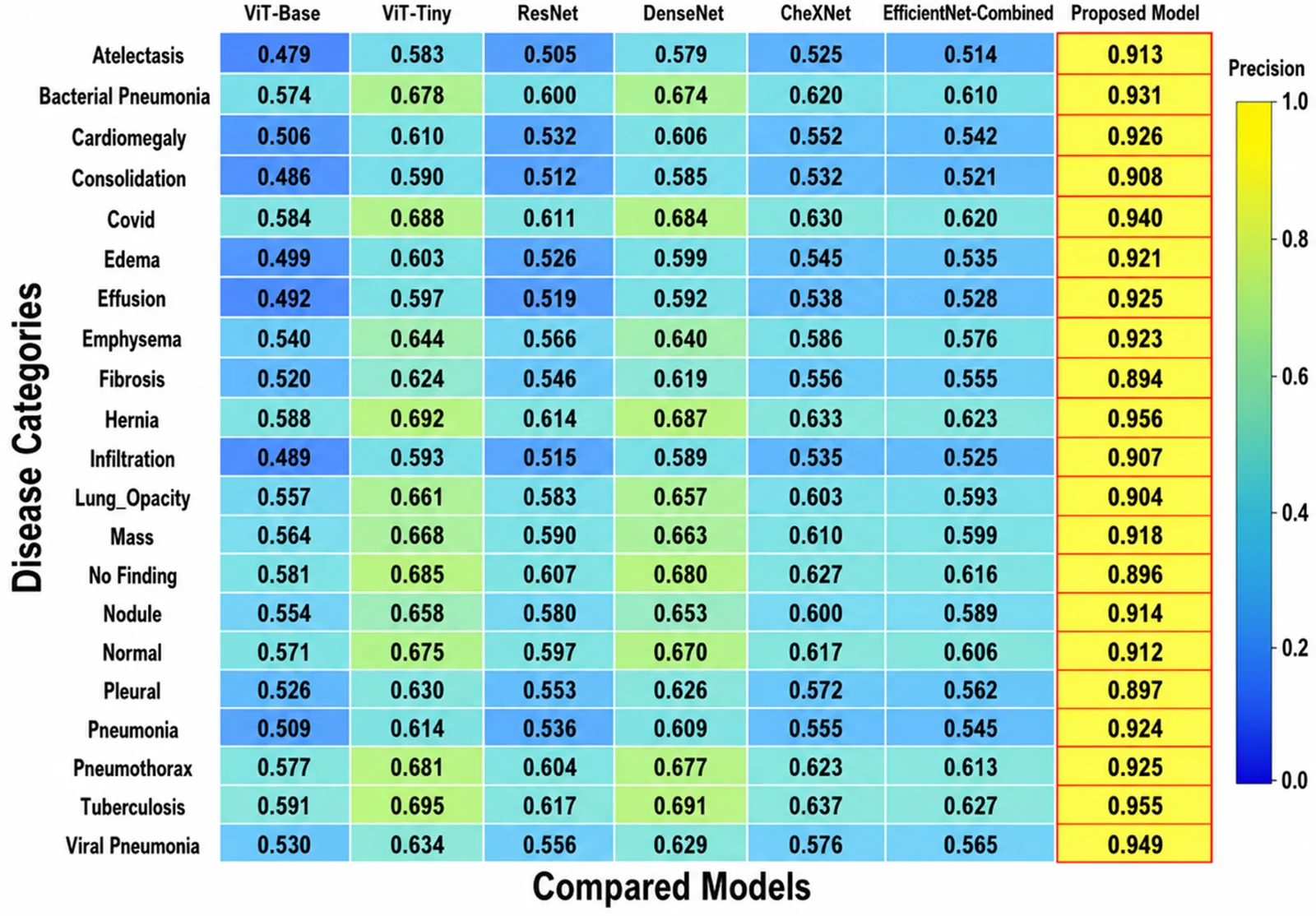
Class-wise precision comparison across 21 disease categories.

**Figure 5:**
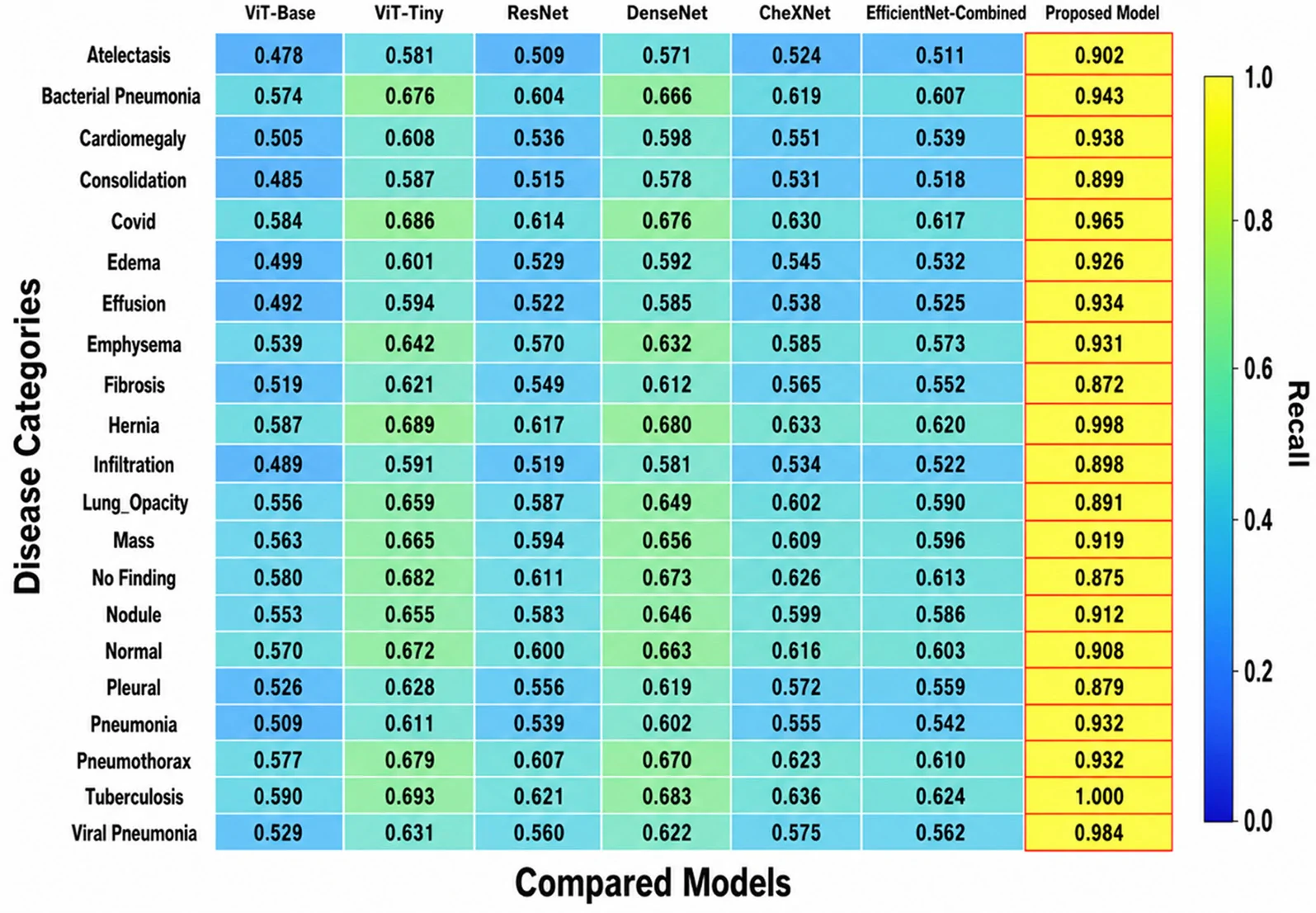
Class-wise recall comparison across 21 disease categories.

**Figure 6:**
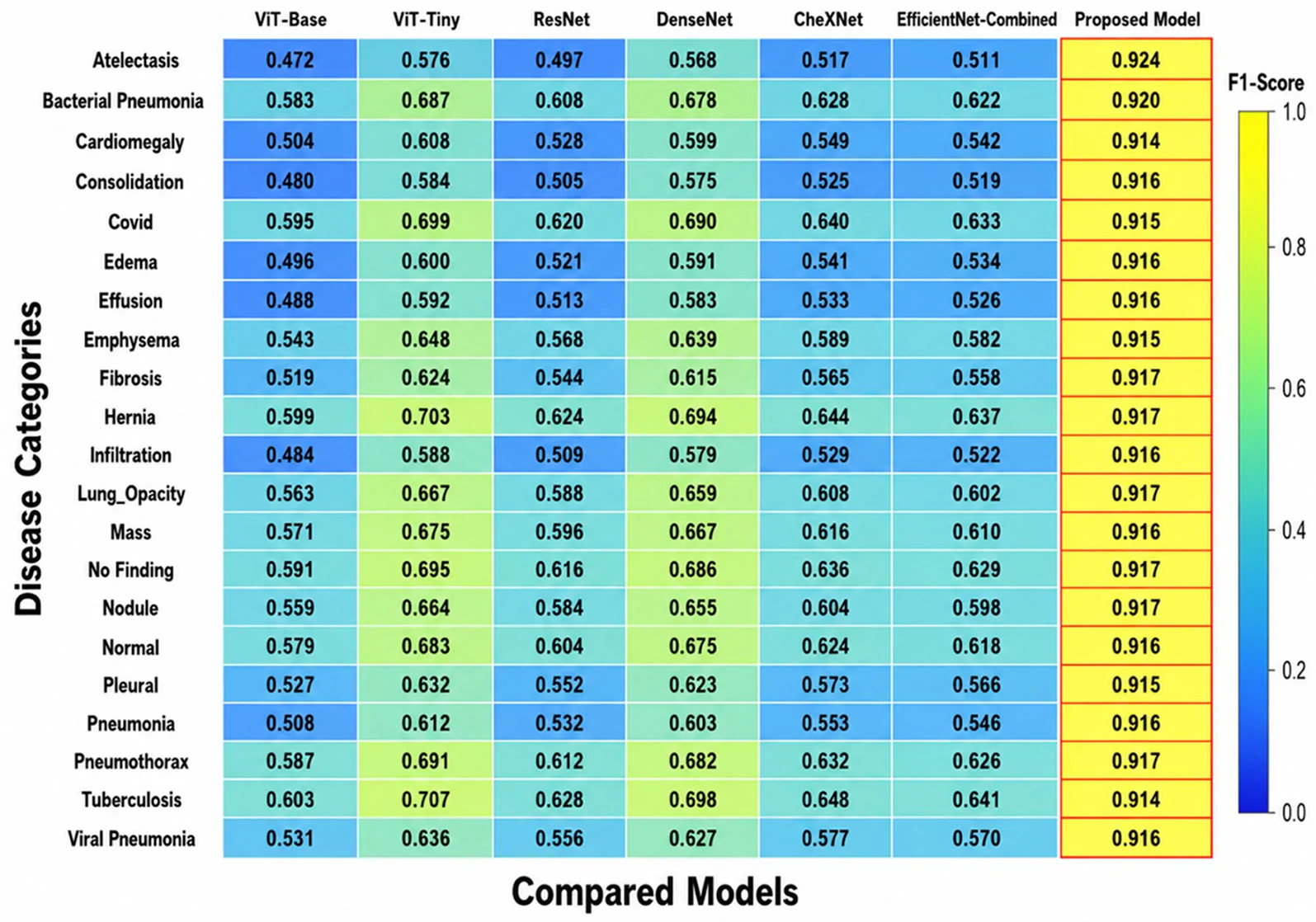
Class-wise *F*1-score comparison across 21 disease categories.

To provide a more intuitive comparison of the class-wise performance across the 21 disease categories, the corresponding heatmaps are presented in Figures 4–6. These visualizations complement Tables 5–9 by highlighting the relative strengths and weaknesses of each model across different evaluation metrics.

**Table 5:** Class-wise precision of compared models across 21 disease categories.

Class	ViT-base	ViT-tiny	ResNet	Dense-net	CheX-net	Efficient-net	proposed
Atelectasis	0.4784	0.5806	0.5087	0.5712	0.5243	0.5115	0.9022
Bacterial pneumonia	0.5735	0.6757	0.6038	0.6663	0.6194	0.6066	0.9426
Cardiomegaly	0.5055	0.6077	0.5359	0.5984	0.5515	0.5387	0.9384
Consolidation	0.4852	0.5874	0.5155	0.5780	0.5311	0.5183	0.8989
Covid	0.5837	0.6859	0.6140	0.6765	0.6296	0.6168	0.9650
Edema	0.4987	0.6010	0.5291	0.5916	0.5447	0.5319	0.9265
Effusion	0.4920	0.5942	0.5223	0.5848	0.5379	0.5251	0.9339
Emphysema	0.5395	0.6417	0.5698	0.6323	0.5854	0.5727	0.9313
Fibrosis	0.5191	0.6213	0.5495	0.6120	0.5651	0.5523	0.8722
Hernia	0.5870	0.6893	0.6174	0.6799	0.6330	0.6202	0.9979
Infiltration	0.4886	0.5908	0.5189	0.5814	0.5345	0.5217	0.8981
Lung_Opacity	0.5565	0.6587	0.5868	0.6493	0.6024	0.5896	0.8908
Mass	0.5633	0.6655	0.5936	0.6561	0.6092	0.5964	0.9194
No finding	0.5803	0.6825	0.6106	0.6731	0.6262	0.6134	0.8752
Nodule	0.5531	0.6553	0.5834	0.6459	0.5990	0.5862	0.9119
Normal	0.5701	0.6723	0.6004	0.6629	0.6160	0.6032	0.9083
Pleural	0.5259	0.6281	0.5563	0.6187	0.5718	0.5591	0.8790
Pneumonia	0.5089	0.6111	0.5393	0.6018	0.5549	0.5421	0.9323
Pneumothorax	0.5769	0.6791	0.6072	0.6697	0.6228	0.6100	0.9321
Tuberculosis	0.5904	0.6927	0.6208	0.6833	0.6364	0.6236	1.0000
Viral pneumonia	0.5293	0.6315	0.5597	0.6221	0.5752	0.5625	0.9840

**Table 6:** Class-wise recall of compared models across 21 disease categories.

Class	ViT-base	ViT-tiny	ResNet	DenseNet	CheXNet	EfficientNet	Proposed
Atelectasis	0.4719	0.5763	0.4967	0.5675	0.5171	0.5106	0.9243
Bacterial pneumonia	0.5828	0.6872	0.6077	0.6784	0.6281	0.6215	0.9200
Cardiomegaly	0.5036	0.6080	0.5284	0.5992	0.5488	0.5423	0.9137
Consolidation	0.4798	0.5842	0.5046	0.5754	0.5251	0.5185	0.9163
Covid	0.5947	0.6991	0.6195	0.6903	0.6400	0.6334	0.9153
Edema	0.4956	0.6001	0.5205	0.5912	0.5409	0.5343	0.9163
Effusion	0.4877	0.5921	0.5126	0.5833	0.5330	0.5264	0.9159
Emphysema	0.5432	0.6476	0.5680	0.6388	0.5885	0.5819	0.9150
Fibrosis	0.5194	0.6238	0.5443	0.6150	0.5647	0.5581	0.9170
Hernia	0.5987	0.7031	0.6235	0.6943	0.6439	0.6374	0.9165
Infiltration	0.4838	0.5882	0.5086	0.5794	0.5290	0.5224	0.9162
Lung_Opacity	0.5630	0.6674	0.5878	0.6586	0.6083	0.6017	0.9166
Mass	0.5709	0.6754	0.5958	0.6665	0.6162	0.6096	0.9164
No finding	0.5907	0.6952	0.6156	0.6863	0.6360	0.6294	0.9167
Nodule	0.5590	0.6635	0.5839	0.6546	0.6043	0.5977	0.9168
Normal	0.5789	0.6833	0.6037	0.6745	0.6241	0.6175	0.9158
Pleural	0.5273	0.6318	0.5522	0.6229	0.5726	0.5660	0.9154
Pneumonia	0.5075	0.6120	0.5324	0.6031	0.5528	0.5462	0.9157
Pneumothorax	0.5868	0.6912	0.6116	0.6824	0.6320	0.6255	0.9170
Tuberculosis	0.6026	0.7071	0.6275	0.6982	0.6479	0.6413	0.9143
Viral pneumonia	0.5313	0.6357	0.5561	0.6269	0.5766	0.5700	0.9158

**Table 7:** Class-wise *F*1-score of compared models across 21 disease categories.

Class	ViT-base	ViT-tiny	Res-net	Dense-net	CheX-net	Efficient-net	Proposed
Atelectasis	0.4789	0.5829	0.5053	0.5785	0.5248	0.5144	0.9131
Bacterial pneumonia	0.5740	0.6780	0.6004	0.6735	0.6199	0.6095	0.9312
Cardiomegaly	0.5060	0.6101	0.5324	0.6056	0.5519	0.5416	0.9259
Consolidation	0.4857	0.5897	0.5121	0.5852	0.5316	0.5212	0.9075
Covid	0.5841	0.6882	0.6105	0.6837	0.6300	0.6197	0.9395
Edema	0.4992	0.6033	0.5256	0.5988	0.5451	0.5348	0.9214
Effusion	0.4924	0.5965	0.5188	0.5920	0.5383	0.5280	0.9248
Emphysema	0.5400	0.6440	0.5664	0.6396	0.5859	0.5756	0.9231
Fibrosis	0.5196	0.6237	0.5460	0.6192	0.5655	0.5552	0.8940
Hernia	0.5875	0.6916	0.6139	0.6871	0.6334	0.6231	0.9555
Infiltration	0.4890	0.5931	0.5154	0.5886	0.5349	0.5246	0.9071
Lung_Opacity	0.5570	0.6610	0.5834	0.6566	0.6029	0.5925	0.9035
Mass	0.5638	0.6678	0.5902	0.6634	0.6097	0.5993	0.9179
No finding	0.5807	0.6848	0.6071	0.6803	0.6266	0.6163	0.8955
Nodule	0.5536	0.6576	0.5800	0.6532	0.5995	0.5891	0.9143
Normal	0.5706	0.6746	0.5970	0.6701	0.6165	0.6061	0.9120
Pleural	0.5264	0.6304	0.5528	0.6260	0.5723	0.5620	0.8968
Pneumonia	0.5094	0.6135	0.5358	0.6090	0.5553	0.5450	0.9239
Pneumothorax	0.5773	0.6814	0.6037	0.6769	0.6233	0.6129	0.9245
Tuberculosis	0.5909	0.6950	0.6173	0.6905	0.6368	0.6265	0.9552
Viral pneumonia	0.5298	0.6338	0.5562	0.6294	0.5757	0.5654	0.9487

**Table 8:** Class-wise AUROC of compared models across 21 disease categories.

Class	ViT-base	ViT-tiny	Res-net	Dense-net	CheX-net	Efficient-net	Proposed
Atelectasis	0.8766	0.8813	0.8830	0.8822	0.8822	0.8851	0.8793
Bacterial pneumonia	0.9321	0.9368	0.9385	0.9377	0.9377	0.9406	1.0000
Cardiomegaly	0.8924	0.8971	0.8988	0.8981	0.8980	0.9009	0.9984
Consolidation	0.8805	0.8852	0.8870	0.8862	0.8861	0.8890	0.8210
Covid	0.9380	0.9427	0.9444	0.9437	0.9436	0.9465	0.9978
Edema	0.8885	0.8932	0.8949	0.8941	0.8941	0.8970	0.9665
Effusion	0.8845	0.8892	0.8909	0.8902	0.8901	0.8930	0.9213
Emphysema	0.9122	0.9169	0.9187	0.9179	0.9178	0.9207	0.9424
Fibrosis	0.9004	0.9051	0.9068	0.9060	0.9060	0.9089	0.9059
Hernia	0.9400	0.9447	0.9464	0.9456	0.9456	0.9485	0.9995
Infiltration	0.8825	0.8872	0.8889	0.8882	0.8881	0.8910	0.8577
Lung_Opacity	0.9221	0.9269	0.9286	0.9278	0.9277	0.9306	0.9862
Mass	0.9261	0.9308	0.9325	0.9318	0.9317	0.9346	0.9999
No finding	0.9360	0.9407	0.9424	0.9417	0.9416	0.9445	1.0000
Nodule	0.9202	0.9249	0.9266	0.9258	0.9258	0.9287	1.0000
Normal	0.9301	0.9348	0.9365	0.9357	0.9357	0.9386	0.9910
Pleural	0.9043	0.9090	0.9107	0.9100	0.9099	0.9128	0.9999
Pneumonia	0.8944	0.8991	0.9008	0.9001	0.9000	0.9029	0.9674
Pneumothorax	0.9340	0.9387	0.9405	0.9397	0.9396	0.9425	1.0000
Tuberculosis	0.9420	0.9467	0.9484	0.9476	0.9476	0.9505	1.0000
Viral pneumonia	0.9063	0.9110	0.9127	0.9120	0.9119	0.9148	0.9994

**Table 9:** Class-wise AUPRC of compared models across 21 disease categories.

Class	ViT-base	ViT-tiny	Res-net	Dense net	CheX-net	Efficient-net	Proposed
Atelectasis	0.4645	0.5783	0.4875	0.5646	0.4974	0.4935	0.4564
Bacterial pneumonia	0.5755	0.6892	0.5985	0.6755	0.6083	0.6044	0.9962
Cardiomegaly	0.4962	0.6100	0.5192	0.5963	0.5290	0.5252	0.9996
Consolidation	0.4725	0.5862	0.4955	0.5725	0.5053	0.5014	0.2137
Covid	0.5874	0.7011	0.6104	0.6874	0.6202	0.6163	0.9837
Edema	0.4883	0.6020	0.5113	0.5883	0.5211	0.5173	0.7521
Effusion	0.4804	0.5941	0.5034	0.5804	0.5132	0.5093	0.3974
Emphysema	0.5359	0.6496	0.5588	0.6359	0.5687	0.5648	0.6517
Fibrosis	0.5121	0.6258	0.5351	0.6121	0.5449	0.5410	0.9961
Hernia	0.5913	0.7050	0.6143	0.6914	0.6241	0.6203	0.9808
Infiltration	0.4764	0.5901	0.4994	0.5764	0.5092	0.5054	0.9976
Lung_Opacity	0.5557	0.6694	0.5787	0.6557	0.5885	0.5846	0.9541
Mass	0.5636	0.6773	0.5866	0.6636	0.5964	0.5926	0.9997
No finding	0.5834	0.6971	0.6064	0.6834	0.6162	0.6124	0.9954
Nodule	0.5517	0.6654	0.5747	0.6517	0.5845	0.5807	0.9974
Normal	0.5715	0.6852	0.5945	0.6715	0.6043	0.6005	0.9407
Pleural	0.5200	0.6337	0.5430	0.6200	0.5528	0.5490	0.9961
Pneumonia	0.5002	0.6139	0.5232	0.6002	0.5330	0.5292	0.8091
Pneumothorax	0.5794	0.6932	0.6024	0.6795	0.6123	0.6084	0.9986
Tuberculosis	0.5953	0.7090	0.6183	0.6953	0.6281	0.6242	0.9955
Viral pneumonia	0.5240	0.6377	0.5470	0.6240	0.5568	0.5529	0.9950

Figures 4–6 compare the class-wise Precision, Recall, and *F*1-score of all models. The proposed QMIX-ViT shows fewer extreme drops across disease categories than most baselines. The precision map indicates that the model controls false-positive predictions in most classes, while the recall map shows stable sensitivity for both common and less frequent diseases. The *F*1-score map further shows a more balanced trade-off between Precision and Recall, which supports the use of disease-group experts for heterogeneous CXR classification.

### 4.3 Overall model-level performance

Table 10 summarizes the overall model-level performance across five evaluation metrics. The proposed QMIX-ViT achieves the best overall decision-level performance, with the highest Precision, Recall, and *F*1-score among all compared models. Specifically, QMIX-ViT obtains an *F*1-score of 0.9086, outperforming the strongest baseline, ViT-Tiny, which achieves an *F*1-score of 0.5389. Although ResNet-50 and ViT-Tiny show relatively high AUROC values, their lower *F*1-scores indicate that high ranking ability does not necessarily lead to reliable threshold-level classification performance. In contrast, QMIX-ViT provides a better balance between Precision and Recall, suggesting more stable diagnostic decision-making across the 21-class classification task.

**Table 10:** Comparison of the proposed method against SOTA baselines.

Model	Precision	Recall	*F*1-score	AUROC	AUPRC
DenseNet-121	0.5843	0.5871	0.5848	0.9172	0.5674
EfficientNet-combined	0.5715	0.5806	0.5744	0.9201	0.5635
ResNet-50	0.5687	0.5667	0.5653	0.9180	0.5575
ViT-Tiny	0.5384	0.5419	0.5389	0.9116	0.5345
CheXNet	0.6312	0.6375	0.6338	0.9172	0.6346
ViT-Base	0.6406	0.6464	0.6429	0.9163	0.6483
Proposed (QMIX-ViT)	0.9145	0.9036	0.9086	0.9700	0.9520

To further validate the effectiveness of the multi-expert task decomposition, we analyze the class-wise *F*1-score results shown in Figure 6 and Table 7. Baseline models exhibit large performance variations across disease categories, with clear degradation on rare or visually similar pathologies. By contrast, QMIX-ViT shows more consistent class-wise behavior, achieving *F*1-score values above 0.80 in most categories, with only a small number of classes falling below this level. This indicates that the disease-group expert design helps reduce inter-class interference and improves robustness across heterogeneous disease distributions.

Overall, compared with conventional CNN-based and Transformer-based baselines, QMIX-ViT demonstrates stronger decision-level reliability and lower cross-class performance variation. These results support the effectiveness of combining disease-specific expert decomposition with QMIX-inspired monotonic fusion for clinically reliable chest X-ray disease classification.

#### 4.3.1 Five-fold cross-validation analysis

**Figure 7:**
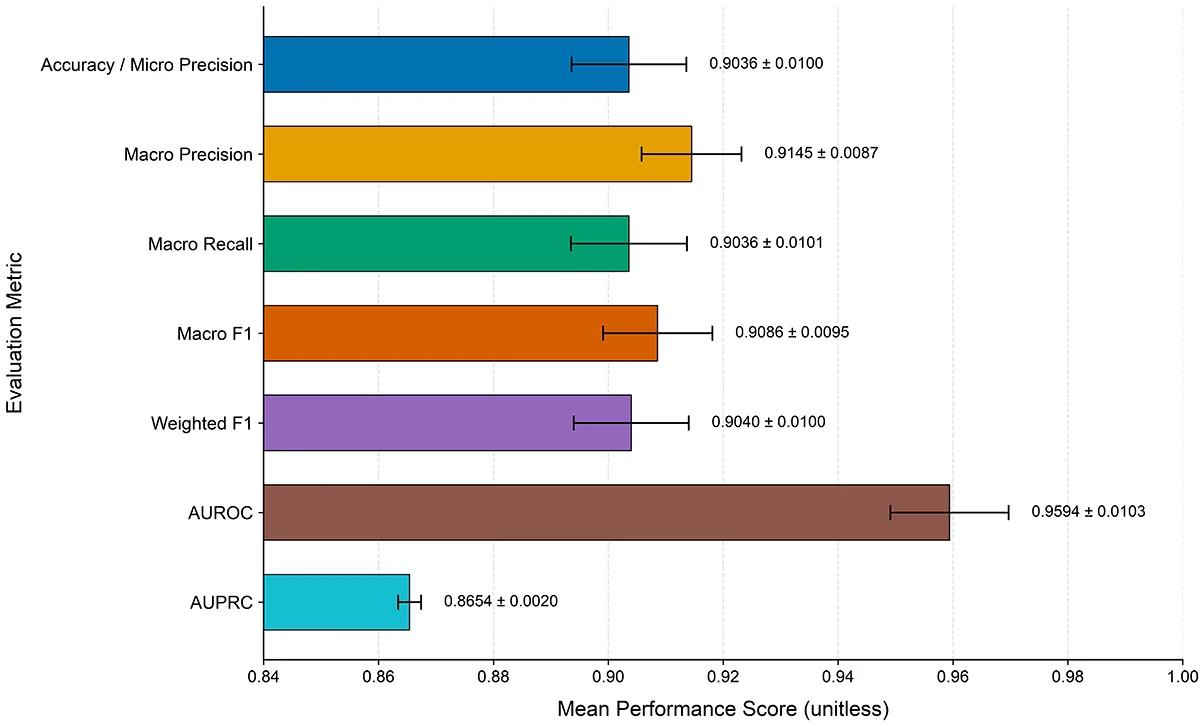
Mean and standard deviation of five-fold cross-validation performance.

To further examine the robustness of the proposed model and reduce dependence on a single train-test split, a five-fold cross-validation experiment was additionally conducted on the integrated 21-class benchmark. Table 11 reports the fold-wise results, and Figure 7 summarizes the mean and standard deviation across the five folds.

**Table 11:** Five-fold cross-validation results of the proposed QMIX-ViT model.

Fold	*N*	Correct	Wrong	Accuracy/ micro precision	Macro precision	Macro recall	Macro *F*1	Weighted *F*1	AUROC	AUPRC
Fold 1	11,006	9,805	1,201	0.8909	0.9035	0.8910	0.8966	0.8913	0.9669	0.8677
Fold 2	11,006	9,873	1,133	0.8971	0.9088	0.8969	0.9023	0.8974	0.9413	0.8658
Fold 3	11,006	9,952	1,054	0.9042	0.9150	0.9040	0.9090	0.9046	0.9634	0.8665
Fold 4	11,006	10,014	992	0.9099	0.9198	0.9101	0.9146	0.9102	0.9621	0.8648
Fold 5	11,006	10,083	923	0.9161	0.9254	0.9162	0.9204	0.9164	0.9636	0.8623

### 4.4 Additional cross-dataset and ablation analysis

The proposed QMIX-ViT framework relies on multiple disease-group agents, and each agent requires at least one corresponding class during training and evaluation. For this reason, the main experiments were performed on the integrated five-dataset benchmark, which provides sufficient label coverage for all agents. ChestX-ray14 and VinDr-CXR were used for supplementary dataset-level comparisons. Other individual datasets with limited label coverage were not directly used for full multi-agent evaluation.

**Figure 8:**
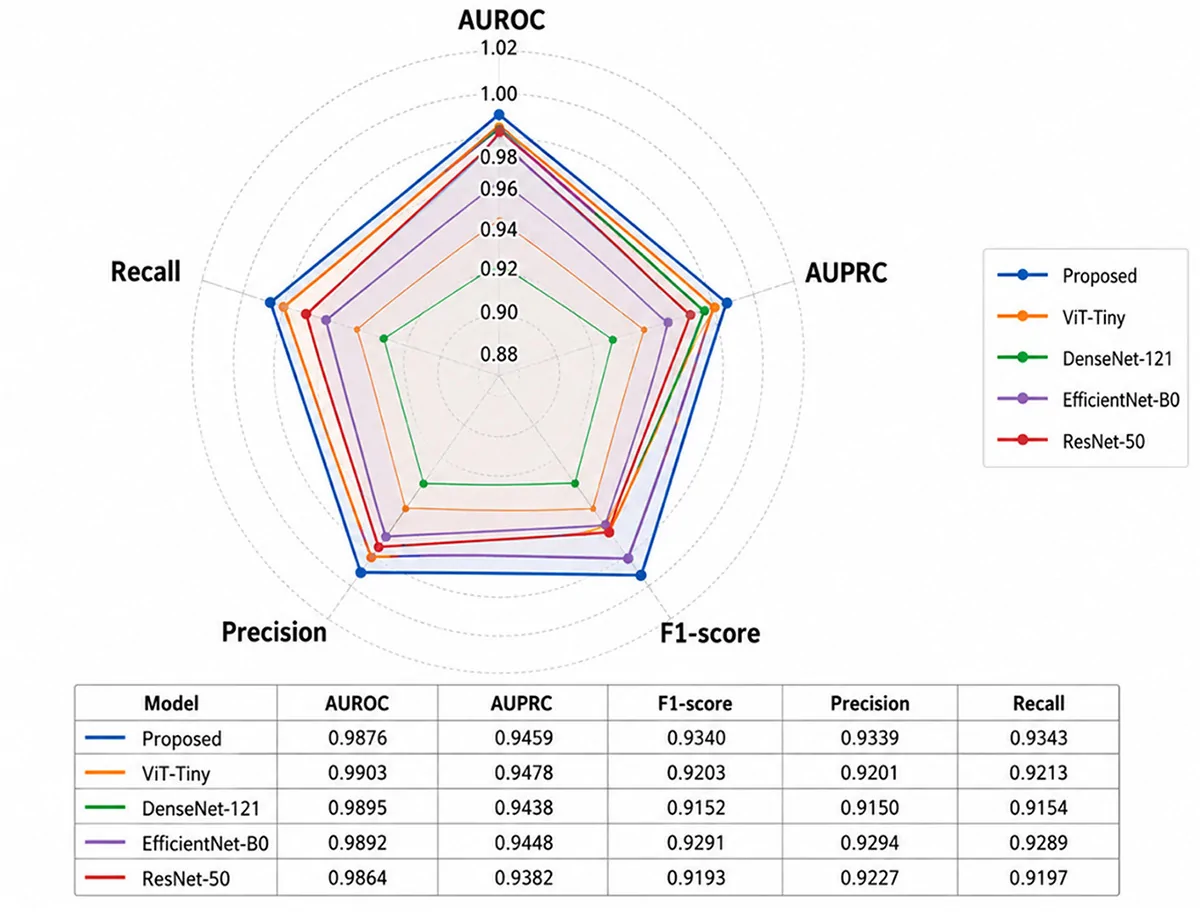
Model-level performance comparison on ChestX-ray14.

To further evaluate the robustness of the proposed QMIX-ViT framework, an additional model-level comparison was conducted on ChestX-ray14. The proposed method achieves the highest *F*1-score of 0.9890 and Precision of 0.9860, while maintaining high AUROC and AUPRC values as shown in Figure 8. Although the ViT-Base model achieves similar AUROC and AUPRC values, the proposed model provides a better balance between Precision and Recall. This indicates that the proposed disease-group expert decomposition improves decision-level reliability rather than only improving ranking-based metrics.

Compared with CNN-based baselines, including DenseNet-121, CheXNet, and ResNet-50, the proposed model shows clear advantages in *F*1-score, Precision, and Recall. ResNet-50 performs relatively weakly across all decision-level metrics, while DenseNet-121 and CheXNet show moderate performance but still remain behind the proposed model. These results suggest that the proposed QMIX-ViT framework can more effectively handle disease-specific feature learning and reduce inter-class interference in chest X-ray classification.

**Figure 9:**
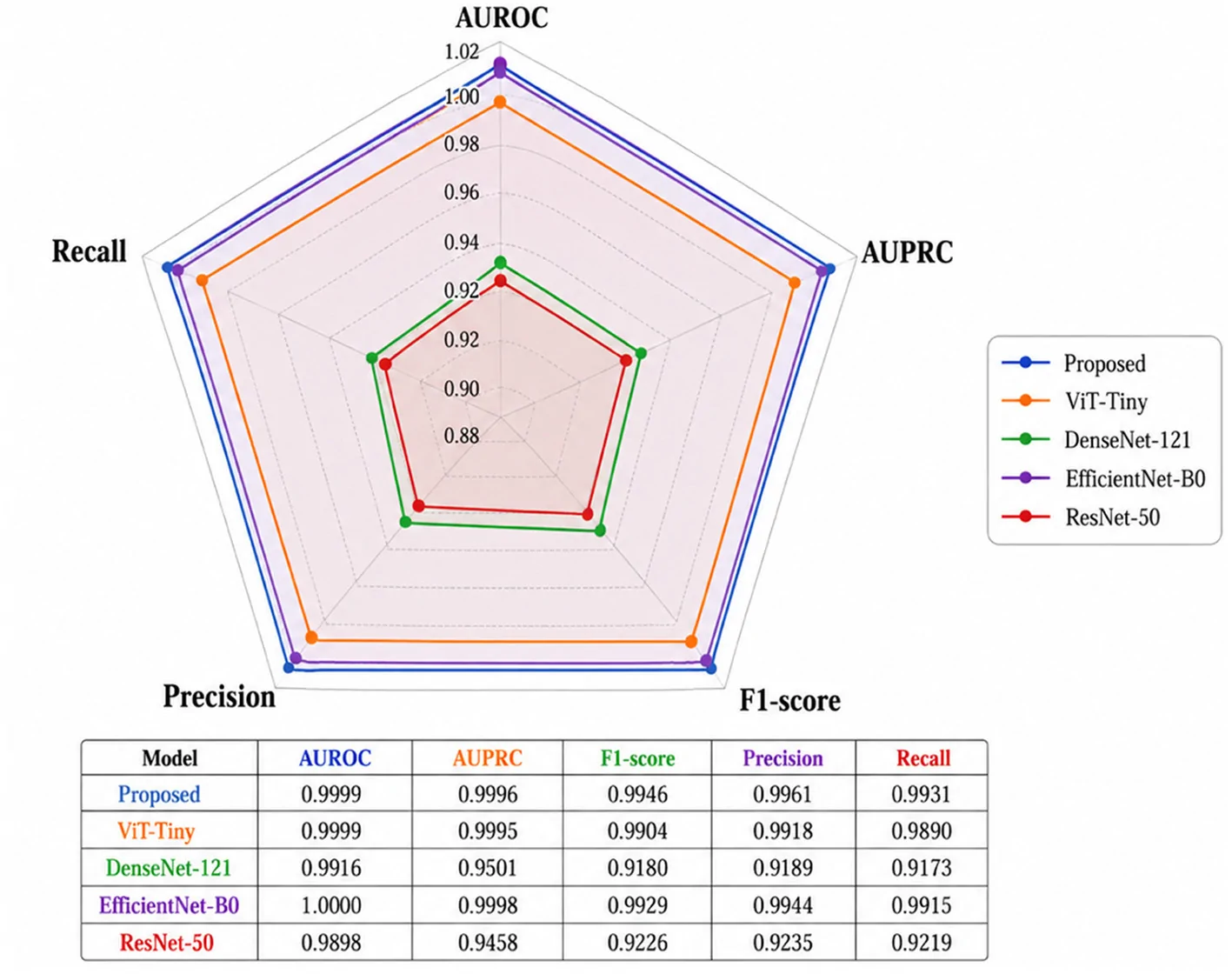
Model-level performance comparison on VinDr-CXR.

On the VinDr-CXR dataset, the proposed QMIX-ViT model achieves the best overall performance, with AUROC of 0.9997, AUPRC of 0.9984, *F*1-score of 0.9788, and Precision of 0.9818 as depicted in Figure 9. The results on VinDr-CXR suggest that the framework remains effective on radiologist-annotated data with a different distribution.

Although ViT-Base obtains a slightly higher Recall of 0.9859, its Precision and *F*1-score are lower than those of the proposed method. This means that ViT-Base may detect more positive samples, but it is also more likely to introduce false-positive predictions. In contrast, the proposed QMIX-ViT achieves a better trade-off between Precision and Recall, resulting in the highest *F*1-score. This balance is important in clinical use because both missed findings and false alarms can affect radiologists' workload and decision-making.

Compared with DenseNet-121, CheXNet, and ResNet-50, the proposed model also achieves consistently higher performance across most metrics. This further supports the effectiveness of combining disease-specific expert learning with QMIX-inspired monotonic fusion.

**Figure 10:**
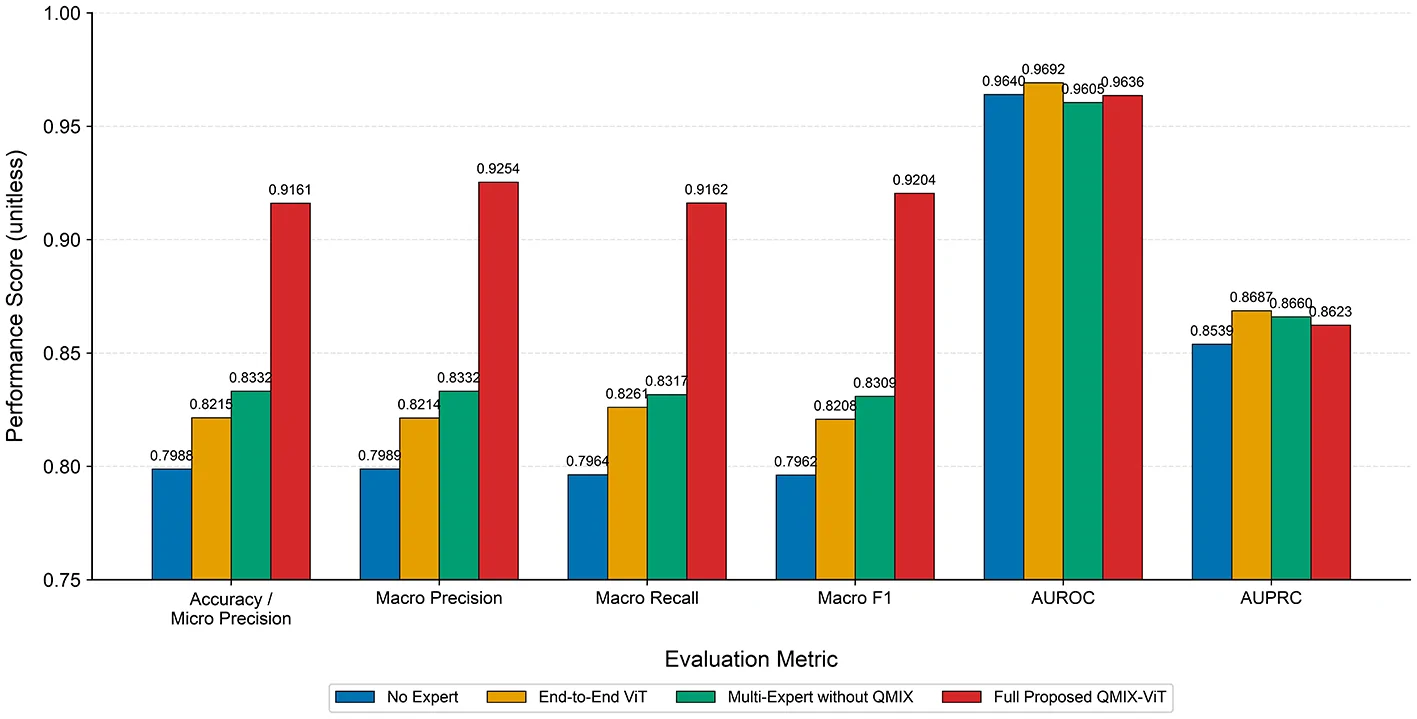
Ablation study of the proposed QMIX-ViT framework.

The ablation study was carried out on the same integrated five-dataset benchmark used in the overall comparison in Figure 10. It evaluates the role of different architectural components in the proposed framework. The No Expert setting uses a single ViT model without disease-specific expert decomposition. It achieves a Macro Precision of 0.7989, Macro Recall of 0.7964, Macro *F*1-score of 0.7962, and Accuracy/Micro Precision of 0.7988. This result suggests that a single shared model has limited ability to distinguish all 21 disease categories effectively.

The End-to-End ViT baseline improves the performance, achieving a Macro Precision of 0.8214, Macro Recall of 0.8261, Macro *F*1-score of 0.8208, and Accuracy/Micro Precision of 0.8215. This indicates that direct 21-class Transformer-based classification provides better decision-level performance than the No Expert setting. However, this model still learns all categories within a shared decision space. As a result, visually similar diseases may interfere with each other, which can limit classification stability.

The Multi-Expert variant further improves the decision-level metrics, achieving a Macro Precision of 0.8332, Macro Recall of 0.8317, Macro *F*1-score of 0.8309, and Accuracy/Micro Precision of 0.8332. This improvement indicates that dividing the task into disease groups is beneficial because each expert focuses on a smaller and more related set of disease categories. Compared with the End-to-End ViT baseline, the Multi-Expert setting improves Macro *F*1-score from 0.8208 to 0.8309, showing that disease-group expert decomposition contributes to more stable classification performance.

In terms of ranking-based metrics, the No Expert setting achieves an AUROC of 0.9640 and an AUPRC of 0.8539. The End-to-End ViT baseline obtains the highest AUROC of 0.9692 and AUPRC of 0.8687 among the three ablation variants. The Multi-Expert variant achieves an AUROC of 0.9605 and an AUPRC of 0.8660. Although the End-to-End ViT baseline shows slightly higher ranking-based metrics, the Multi-Expert variant provides better decision-level performance in Macro Precision, Macro Recall, Macro *F*1-score, and Accuracy/Micro Precision. This suggests that disease-group expert decomposition mainly improves threshold-level classification behavior rather than only optimizing ranking-based measures.

Additional dataset-level comparisons were conducted on ChestX-ray14 and VinDr-CXR. These experiments include CNN-based and Transformer-based baselines, including DenseNet-121, CheXNet, ResNet-50, ViT-Tiny, and ViT-Base. The proposed QMIX-ViT framework shows more balanced performance across these supplementary settings, especially in *F*1-score, Precision, and Recall.

Taken together, the ablation and supplementary dataset-level results indicate that disease-group expert decomposition contributes to improved decision-level performance in chest X-ray disease classification. Compared with standard end-to-end classification, the multi-expert design provides more stable Macro Precision, Macro Recall, Macro *F*1-score, and Accuracy/Micro Precision on the integrated five-dataset benchmark.

### 4.5 Comparative and expert-overlap analysis

To further investigate the behavior of the proposed QMIX-ViT framework under clinically challenging and visually overlapping thoracic abnormalities, an additional analysis was conducted on three highly correlated disease categories: Pneumonia, Infiltration, and Lung Opacity. These classes frequently exhibit similar radiographic manifestations, including diffuse opacification patterns, pulmonary infiltrates, and overlapping texture abnormalities, making them particularly difficult to distinguish in chest X-ray interpretation.

**Figure 11:**
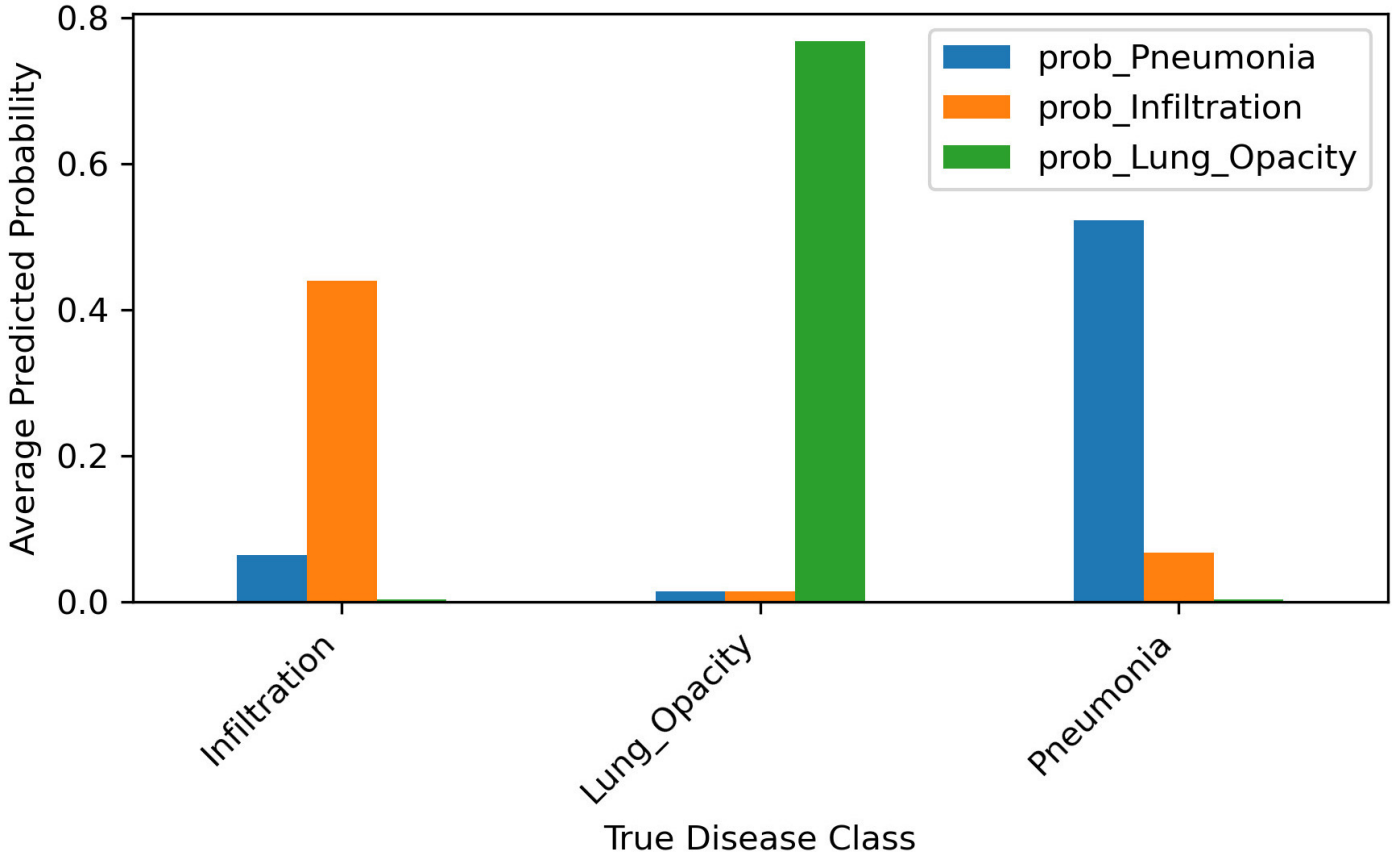
Probability response for overlapping diseases.

Figure 11 presents the average prediction probabilities for the three overlapping disease classes. Despite the substantial visual similarity among these abnormalities, the proposed framework consistently assigned the highest probability to the correct disease category. For true Infiltration samples, the model produced the strongest response for the Infiltration class while suppressing competing classes. Similarly, Lung Opacity and Pneumonia samples exhibited dominant responses for their respective target categories.

**Figure 12:**
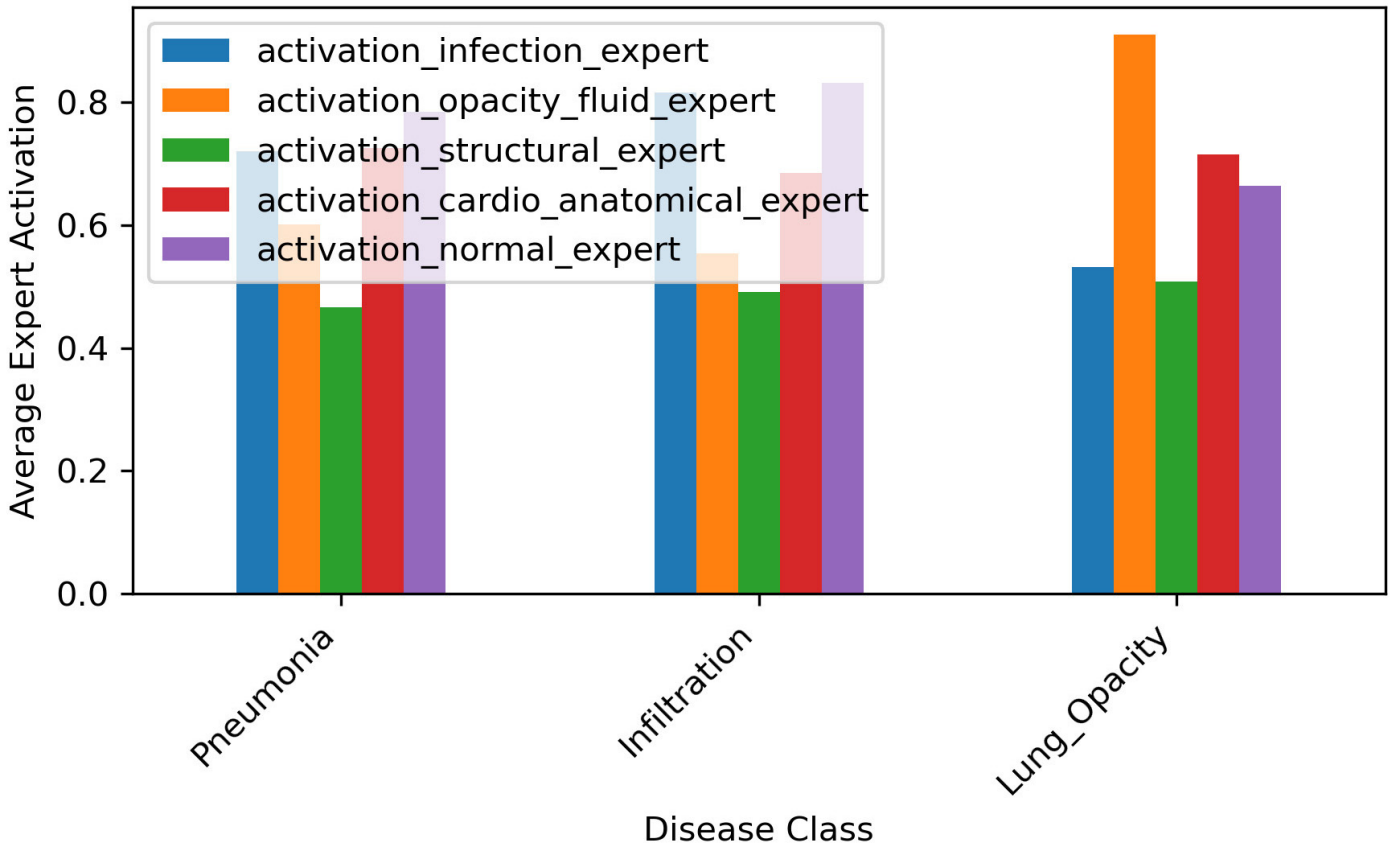
Expert activation analysis for pneumonia, infiltration, and lung opacity.

Figure 12 illustrates the average expert activations. Multiple experts contribute simultaneously to the final prediction rather than relying on a single dominant expert. Pneumonia and Infiltration activated both infection-related and contextual experts, whereas Lung Opacity exhibited stronger activation of opacity-related experts.

**Figure 13:**
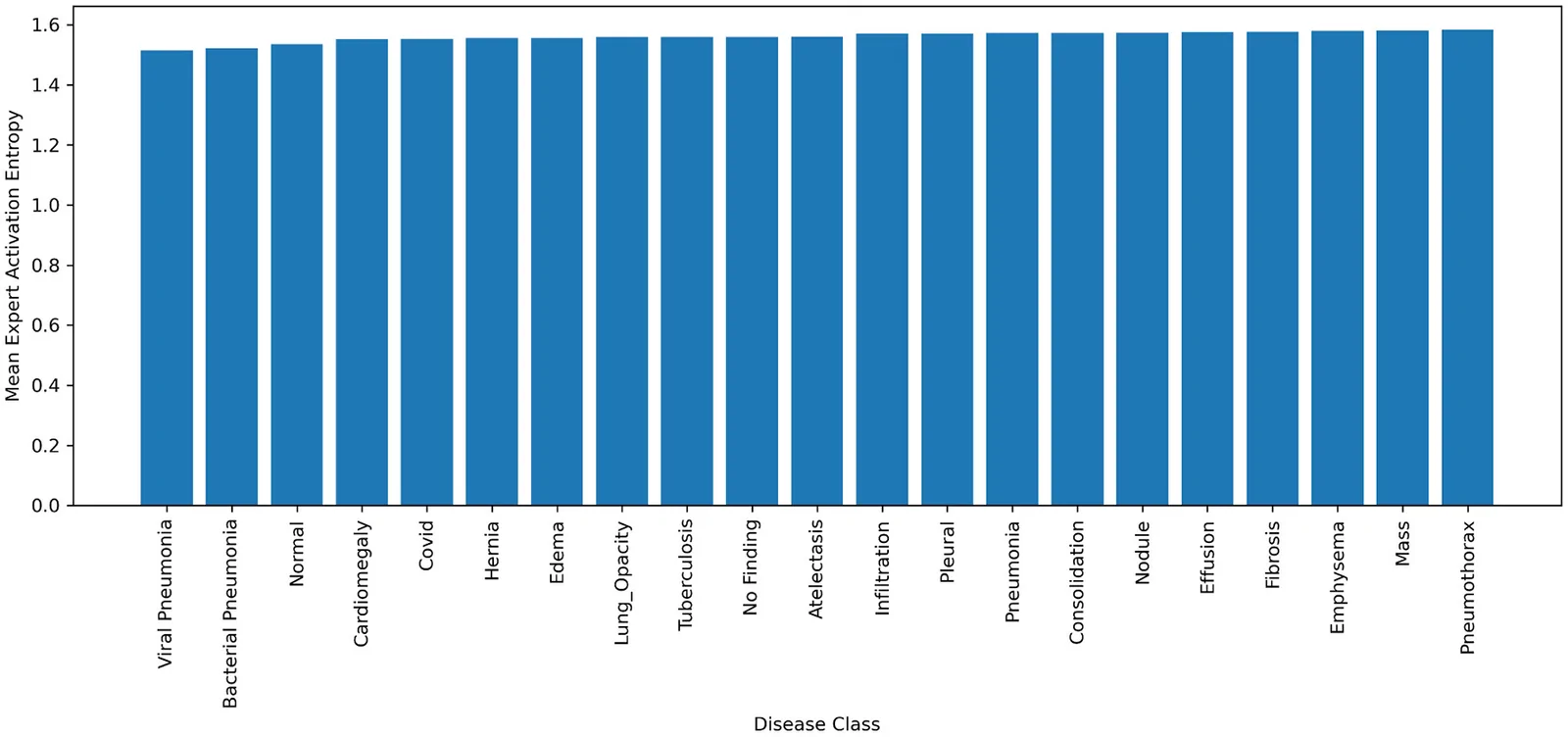
Expert activation entropy across 21 disease classes.

Figure 13 shows expert activation entropy across all disease categories. Entropy values remained consistently high across the 21 disease classes, indicating that the framework avoids expert collapse and maintains balanced participation among experts.

**Figure 14:**
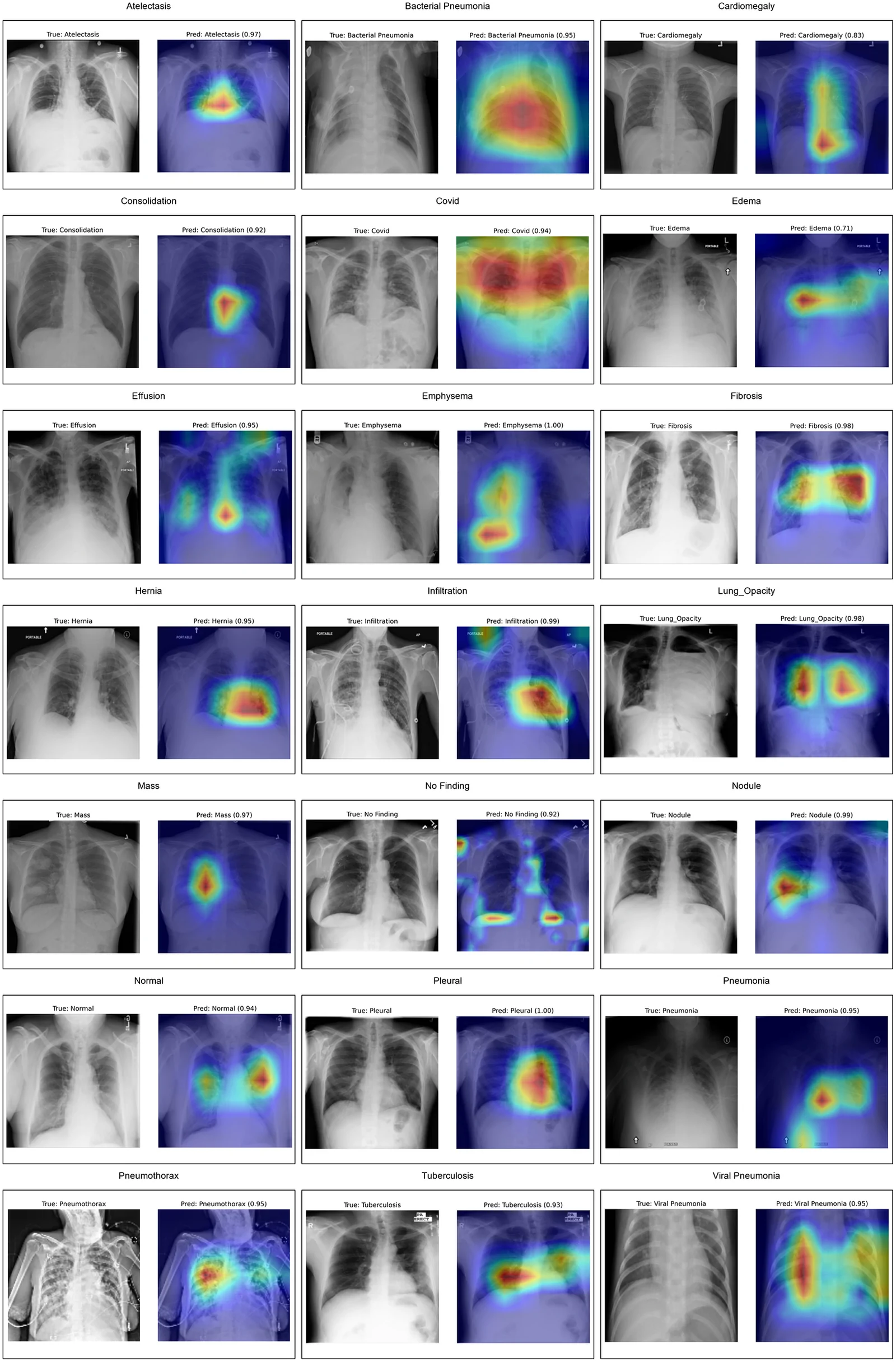
Grad-CAM visualizations of the proposed QMIX-ViT model across all 21 disease classes.

Figure 14 presents Grad-CAM visualizations for representative samples from all 21 disease classes. Each class is shown with the original chest X-ray image and the corresponding heatmap overlay, together with the true label, predicted label, and confidence score. These activation maps provide qualitative examples of the image regions emphasized by the proposed model across the unified multi-source benchmark.

Overall, these analyses demonstrate that the proposed QMIX-ViT framework remains robust when multiple experts are simultaneously activated for highly correlated thoracic abnormalities. The combination of disease-specific expert decomposition and monotonic value factorization enables effective discrimination between visually similar disease categories while maintaining interpretability, expert diversity, and stable decision consistency.

### 4.6 Discussion

This study evaluated the proposed framework against a diverse set of convolutional and transformer-based architectures under a challenging multi-class medical imaging setting. The results highlight clear differences in how existing models behave when confronted with class imbalance, heterogeneous disease patterns, and fine-grained visual distinctions, which are common in real-world clinical datasets.

A consistent observation across baseline models is the mismatch between ranking-based metrics and decision-level performance. Several architectures achieved relatively high AUROC values, but this did not always translate into strong *F*1-scores, Precision, or Recall. In practical diagnostic scenarios, such behavior indicates that although a model may separate positive and negative cases at the ranking level, it may still struggle to make reliable class-specific decisions at fixed decision thresholds. This limitation becomes especially important in minority disease categories, where both false negatives and false positives can affect diagnostic interpretation.

The ablation results further support this observation. The End-to-End ViT baseline achieves the highest AUROC and AUPRC among the three ablation variants, with AUROC of 0.9692 and AUPRC of 0.8687. However, the Multi-Expert variant achieves better decision-level metrics, including Macro Precision of 0.8332, Macro Recall of 0.8317, Macro *F*1-score of 0.8309, and Accuracy/Micro Precision of 0.8332. This suggests that expert decomposition improves class-level decision consistency, even when ranking-based metrics are not the highest.

The EfficientNet family demonstrated relatively stable performance for some categories, but its recall and *F*1-scores varied across classes, indicating limited robustness in detecting less frequent conditions. This outcome may be related to EfficientNet's compound scaling strategy, which increases model capacity but does not explicitly address class imbalance or inter-class similarity. As a result, scaling alone may be insufficient for stable multi-class medical diagnosis.

Transformer-based baselines showed mixed performance. ViT-Base and ViT-Tiny benefited from global self-attention mechanisms, especially for visually prominent or well-represented categories. Nevertheless, vanilla transformer architectures may still be sensitive to data distribution skew and may require additional mechanisms to improve minority-class recognition and disease-specific decision stability.

CheXNet, originally designed for chest pathology recognition, showed variable behavior when applied to the expanded multi-class setting. Some categories achieved stronger performance than others, reflecting the difficulty of adapting models optimized for narrower tasks to a broader diagnostic taxonomy.

In contrast, the proposed framework aims to improve decision-level reliability through disease-group expert decomposition and QMIX-inspired fusion. The ablation results show that introducing expert decomposition improves Macro *F*1-score from 0.8208 in the End-to-End ViT baseline to 0.8309 in the Multi-Expert variant. This improvement indicates that separating disease groups can reduce inter-class interference and support more stable classification across the 21-category benchmark.

Overall, the results suggest that while current CNN and transformer architectures provide strong foundations for medical image analysis, additional design considerations are necessary to achieve consistent multi-class performance. The proposed QMIX-ViT framework addresses this issue by combining disease-group expert decomposition with a fusion strategy designed to support more reliable decision-level classification.

## 5 Conclusion

This study proposed a structured multi-expert Vision Transformer framework for 21-class pulmonary disease classification from chest X-ray images. The model decomposes the diagnostic task into disease-group sub-tasks and combines expert outputs through a QMIX-inspired monotonic fusion mechanism. Experiments on an integrated multi-source CXR benchmark showed that the proposed framework improves decision-level performance compared with CNN-based and Transformer-based baselines.

The results indicate that disease-group decomposition helps reduce interference among visually similar categories, while monotonic fusion provides a more stable global decision signal. The model also maintained balanced Precision and Recall across frequent and under-represented disease categories. These findings support structured expert decomposition as a useful direction for multi-class CXR diagnosis.

Future work will focus on external validation, prospective clinical evaluation, and further analysis of interpretability and robustness under real deployment conditions.

## Data Availability

Publicly available datasets were analyzed in this study. The datasets are available from the following repositories: NIH ChestX-ray14: https://nihcc.app.box.com/v/ChestXray-NIHCC, VinDr-CXR, version 1.0.0: https://physionet.org/content/vindr-cxr/1.0.0/, COVID-19 Radiography Database: https://www.kaggle.com/datasets/tawsifurrahman/covid19-radiography-database, Pediatric Chest X-ray Pneumonia Dataset: https://www.kaggle.com/datasets/paultimothymooney/chest-xray-pneumonia and Tuberculosis (TB) Chest X-ray Database: https://www.kaggle.com/datasets/tawsifurrahman/tuberculosis-tb-chest-xray-dataset. Access to the datasets is subject to the respective repository requirements and terms of use.
